# Biological and physical controls in the Southern Ocean on past millennial-scale atmospheric CO_2_ changes

**DOI:** 10.1038/ncomms11539

**Published:** 2016-05-17

**Authors:** Julia Gottschalk, Luke C. Skinner, Jörg Lippold, Hendrik Vogel, Norbert Frank, Samuel L. Jaccard, Claire Waelbroeck

**Affiliations:** 1 Godwin Laboratory for Palaeoclimate Research, Earth Sciences Department, University of Cambridge, Downing Street, Cambridge CB2 3EQ, UK; 2 Institute of Geological Sciences and Oeschger Center for Climate Change Research, University of Bern, Baltzerstr. 1-3, Bern 3012, Switzerland; 3 Institute of Environmental Physics, University of Heidelberg, Im Neuenheimer Feld 229, Heidelberg 69120, Germany; 4 Laboratoire des Sciences du Climat et de l'Environnement, LSCE/IPSL, CNRS-CEA-UVSQ, Université de Paris-Saclay, Domaine du CNRS, bât. 12, Gif-sur-Yvette 91198, France

## Abstract

Millennial-scale climate changes during the last glacial period and deglaciation were accompanied by rapid changes in atmospheric CO_2_ that remain unexplained. While the role of the Southern Ocean as a 'control valve' on ocean–atmosphere CO_2_ exchange has been emphasized, the exact nature of this role, in particular the relative contributions of physical (for example, ocean dynamics and air–sea gas exchange) versus biological processes (for example, export productivity), remains poorly constrained. Here we combine reconstructions of bottom-water [O_2_], export production and ^14^C ventilation ages in the sub-Antarctic Atlantic, and show that atmospheric CO_2_ pulses during the last glacial- and deglacial periods were consistently accompanied by decreases in the biological export of carbon and increases in deep-ocean ventilation via southern-sourced water masses. These findings demonstrate how the Southern Ocean's 'organic carbon pump' has exerted a tight control on atmospheric CO_2_, and thus global climate, specifically via a synergy of both physical and biological processes.

## Abstract (web summary)

Direct proxy evidence for the many explanations for past millennial-scale atmospheric CO_2_ changes remains scarce. Here, the authors show that changes in the efficiency of respired carbon storage in the deep Southern Ocean were linked to variations in atmospheric CO_2_ over the last 65,000 years.

## Graphical abstract

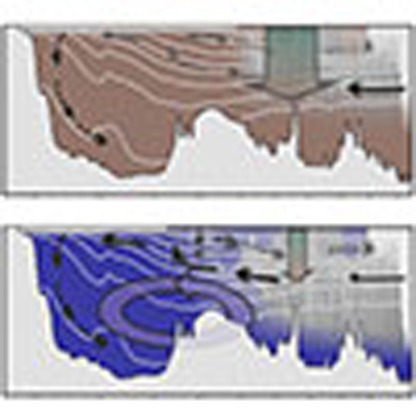

The Southern Ocean is believed to play a key role in the global carbon cycle and millennial-scale variations in atmospheric CO_2_ (CO_2,atm_), which in turn may amplify the impacts of longer-term external climate forcing on global climate^1^. This role stems from the unique control the Southern Ocean is thought to exert on ocean–atmosphere CO_2_ exchange^1^,^2^,^3^ by both facilitating the upward transport of nutrient- and CO_2_-rich water masses along outcropping density surfaces and their exposure to the atmosphere, and modulating the export of biologically fixed carbon into the ocean interior, where it is remineralized and may be effectively isolated from the atmosphere. It has been proposed that these two key aspects of the Southern Ocean's role in the marine carbon cycle may have exerted a dominant control on past CO_2,atm_ change, for instance via variations of dust-driven biological carbon fixation in the sub-Antarctic^4^, the extent of circum-Antarctic sea ice^5^ impeding effective air–sea gas equilibration^6^, and/or changes in the strength or position of southern hemisphere westerlies driving the residual overturning circulation in the Southern Ocean^7^,^8^.

While all of these mechanisms for past CO_2,atm_ change are compelling, observational evidence that might constrain the extent to which they have operated, in particular the balance of biological versus physical (that is, air–sea gas exchange or ocean dynamical) impacts, remains ambiguous. In the sub-Antarctic Atlantic north of the Polar Front (PF), decreased biological export production, along with a diminished aeolian supply of dust (and by inference iron) to the surface ocean, has been found to parallel millennial-scale increases in CO_2,atm_. These observations suggest a significant impact of dust-driven variations of the strength of the ‘organic carbon pump' on CO_2,atm_ (refs 9, 10, 11, 12). However, marked increases in CO_2,atm_ are also accompanied by enhanced export productivity south of the PF (ref. 7). Polar- and sub-polar Southern Ocean export productivity changes thus appear to have opposed each other, raising questions concerning the overall magnitude and sign of the impact of Southern Ocean ‘organic carbon pump' on CO_2,atm_, when integrated across both regions^11^,^13^. On the other hand, while ^14^C evidence has provided direct support for a link between Southern Ocean carbon sequestration (and millennial-scale CO_2,atm_ variability) and physical/dynamical controls on air–sea CO_2_ exchange^14^, these data remain sparse and only extend across the last deglaciation.

Here we present sub-millennially resolved qualitative and quantitative proxy reconstructions of bottom-water [O_2_] from sub-Antarctic Atlantic sediment core MD07-3076Q (14°13.7′W, 44°9.2′S, 3,770 m water depth; Fig. 1) to estimate the apparent oxygen utilization (AOU) in deep waters, which is closely (stoichiometrically) related to the amount of remineralized dissolved inorganic carbon (DIC) because of the consumption of oxygen during the degradation of organic carbon. We use two independent proxy approaches: first, we determined the redox-sensitive enrichment of uranium and manganese in authigenic foraminifer coatings^15^, and second, we measured the difference in carbon isotopic composition between pore waters at the zero-oxygen boundary and overlying bottom waters, which is assumed to be reflected in δ^13^C of the benthic foraminifer *Globobulimina affinis* and *Cibicides kullenbergi*, respectively (Δδ^13^C_*C. kullenbergi–G. affinis*_; refs 16, 17). Our deep sub-Antarctic Atlantic [O_2_] reconstructions show a close correlation to CO_2,atm_ variations during the last deglacial- and glacial periods. The combination of our [O_2_] reconstructions with analyses of ^230^Th-normalized opal fluxes, an indicator of biological export production^7^,^18^, and deep water ^14^C ventilation ages, along with a robust age model for our study core^14^,^19^,^20^ (Methods), highlights that carbon sequestration changes in the southern high latitudes cannot be attributed solely to changes in local biological export production. Instead, they involve significant changes in Southern Ocean vertical mixing and air–sea gas exchange, having direct implications for millennial-scale CO_2,atm_ variations, since 65,000 years before present (BP).

## Results

### Redox-sensitive U and Mn enrichment in foraminifer coatings

The uranium to calcium ratio in authigenic (that is, *in situ* generated) coatings (c), proposed to vary with changes in sedimentary redox-conditions, and therefore with bottom-water [O_2_] (ref. 15), has been measured on weakly chemically cleaned (‘host') calcium carbonate (cc) shells (hereafter referred to as U/Ca_cc+c_) of the planktonic foraminifer *G. bulloides* and the benthic foraminifer *Uvigerina* spp. (Methods). The uranium concentration in the authigenic coatings of foraminifera strongly exceeds the concentration in the foraminiferal shell matrix^21^,^22^. Thus, the overall U/Ca_cc+c_ variability is marginally influenced by the uranium concentration in foraminifer shells, and has been proposed to primarily reflect coating-bound uranium variations instead that is inversely correlated with bottom-water oxygenation^15^. The co-variation of shell weights and U/Ca_cc+c_ levels of *G. bulloides*, however, indicates that shell size and/or wall thickness variations may bias U/Ca_cc+c_ ratios, via changes in the shell mass to surface-area ratio for example (Supplementary Fig. 1). The normalization of coating-bound uranium levels to manganese concentrations circumvents this bias for two reasons: manganese has generally an opposing redox-behaviour to that of uranium^23^,^24^,^25^, and in particular manganese in weakly chemically cleaned foraminiferal tests mainly occurs in Fe-Mn-rich oxyhydroxides and/or Mn-rich carbonate overgrowths attached to the foraminiferal shell^22^,^26^, which may be supported by the observed co-variation of Fe/Ca_cc+c_ and Mn/Ca_cc+c_ levels of *G. bulloides* (Supplementary Fig. 1). We propose that the U/Mn ratio of authigenic coatings in planktonic and benthic foraminifera, U/Mn_c_, serves as reliable indicator of redox-conditions in marine sediments independent of shell matrix variations. The close agreement of planktonic and benthic foraminifer U/Mn_c_ suggests that it sensibly tracks early diagenetic redox-processes within the sediment consistent with previous findings^15^ (Supplementary Fig. 2).

**Figure 1:**
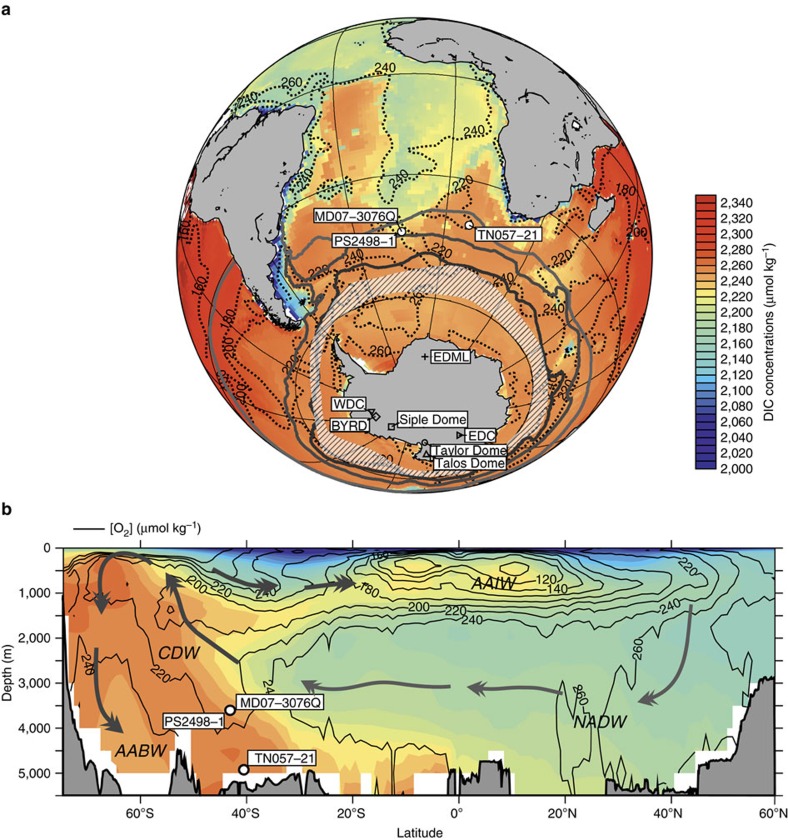
Modern ocean DIC and oxygen concentrations. DIC levels (shaded) and [O_2_] (contours, in μmol kg^−1^)^33^,^63^ in (**a**) Southern Ocean- and Atlantic Ocean bottom waters and (**b**) in a meridional transect across the Atlantic (averaged between 70°W and 20°E). Hatched area broadly represents the region, where the deep DIC reservoir directly ‘communicates' with the surface ocean and the atmosphere along steep density surfaces (equivalent to the area of strong positive CO_2_ fluxes across the air–sea interface in austral winter in the Southern Ocean^64^), which is unique in the global ocean today. White circles show study cores and open symbols mark the location of ice cores that document past changes in atmospheric CO_2_ (CO_2,atm_; as in Figs 2 and 3). Thick lines show the modern positions of the PF, the sub-Antarctic Front (SAF) and the sub-Tropical Front (STF) (south to north)^65^. Arrows show general pathways of North Atlantic Deep Water (NADW), AABW (Antarctic Bottom Water), CDW (Circumpolar Deep Water) and Antarctic Intermediate Water (AAIW).

**Figure 2:**
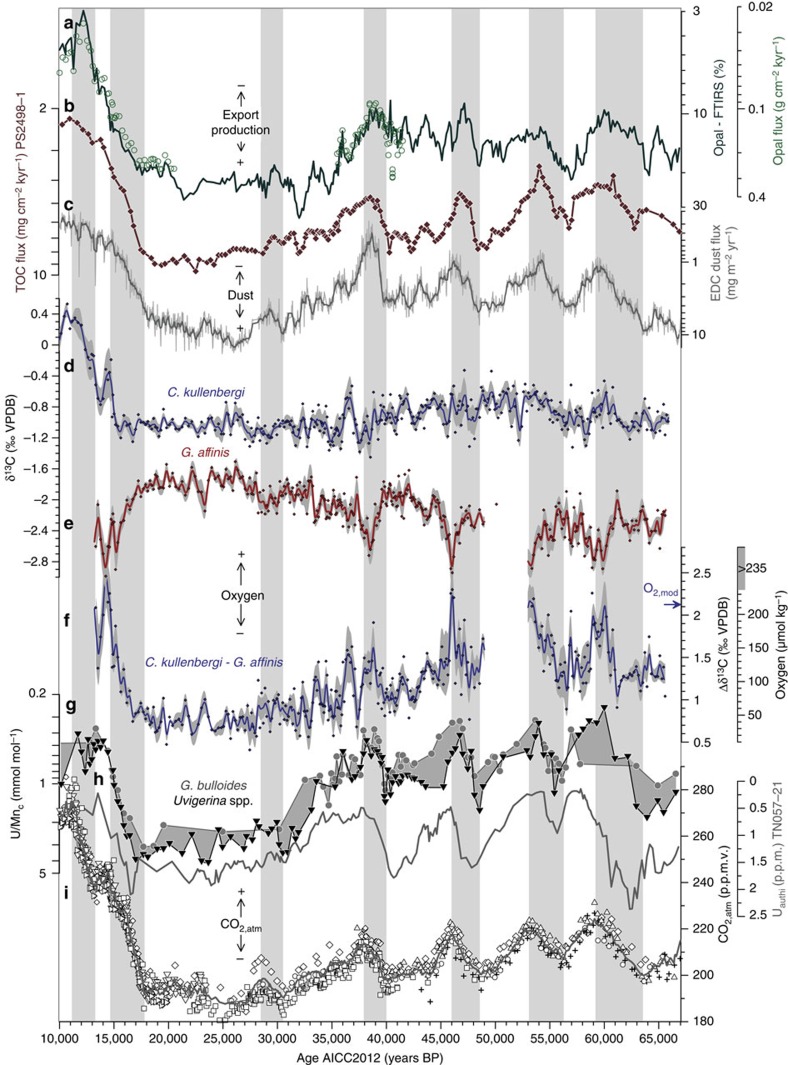
Sub-Antarctic Atlantic bottom-water [O_2_] and productivity changes during the last deglacial and glacial periods. (**a**) Sedimentary opal content (line) and ^230^Thorium-normalized opal fluxes (circles), (**b**) flux of TOC in PS2498-1 (ref. 9; age scale adjusted as outlined in Methods), (**c**) Antarctic (EDC ice core) dust fluxes^66^, (**d**) *C. kullenbergi* δ^13^C (versus Vienna Pee Dee Belemnite (VPDB) standard), (**e**) *G. affinis* δ^13^C (versus VPDB), (**f**) Δδ^13^C_*C. kullenbergi*–*G. affinis*_ and corresponding bottom-water [O_2_] (ref. 16), arrow shows modern [O_2_] at the core site^33^, (**g**) *G. bulloides* (circles) and *Uvigerina* spp. (triangles) U/Mn_c_, (**h**) authigenic uranium concentrations in TN057-21 (ref. 27), (**i**) CO_2,atm_ variations recorded in the Antarctic ice cores BYRD (diamonds)^67^,^68^, EDML (crosses)^47^,^69^, EDC (right-pointed triangles)^70^, Siple Dome (squares)^71^, Talos Dome (triangles)^47^, Taylor Dome (circles)^72^ and WDC (inverted triangles)^73^. All data refer to the AICC2012 age scale^19^,^62^. Lines in **d**–**f** show 500 year-running averages with envelopes indicating the 500 year-window one-sigma standard deviation. Grey bars indicate periods of rising CO_2,atm_.

During the last glacial period, *G. bulloides* and *Uvigerina* spp. U/Mn_c_ are both found to vary with changes in CO_2,atm_ (Fig. 2). During the last deglaciation, the large early deglacial decrease in U/Mn_c_ is clearly synchronous with the initial increase in CO_2,atm_ before 15 kyr BP, while the second pulse in U/Mn_c_ in time with the CO_2,atm_ increase during the following Antarctic warming period (that is, the northern-hemisphere Younger Dryas period) is more equivocal (Fig. 2). Our data are also in good agreement with changes in the authigenic enrichment of uranium in bulk sediments of Cape Basin core TN057-21 (ref. 27; location in Fig. 1), applying the most recent chronology of ref. 28 (Fig. 2). This suggests a basin-wide relevance of observed changes in sedimentary redox-conditions in the central sub-Antarctic Atlantic for variations in CO_2,atm_.

### Benthic foraminifer δ^13^C gradients and bottom-water [O_2_]

Redox-conditions in marine sediments generally reflect changes in organic carbon respiration within the sediment modulated by the downward diffusion of oxygen from bottom waters and/or the organic carbon supply to the sea floor^29^. Aerobic degradation of organic matter is the most efficient pathway of the respiration of organic carbon. Most of organic matter respiration therefore occurs above the sedimentary anoxic boundary. At the anoxic boundary, the diffusion of oxygen from the bottom water into the sediment is balanced by the rate of oxygen consumption during aerobic sedimentary organic carbon respiration in the sub-surface sediment column, such that [O_2_] becomes zero. As organic carbon has typical δ^13^C values of about −22 ‰, the release of ^13^C-depleted carbon during the degradation of organic matter substantially drives the δ^13^C gradient in marine sub-surface pore waters^30^. The total amount of aerobic sedimentary organic carbon respiration is thus a function of bottom-water [O_2_] and is reflected in the δ^13^C difference between bottom waters and pore waters at the zero-oxygen boundary^16^,^30^.

The deep infaunal foraminifer *G. affinis* actively chooses the low-oxygen microhabitat near or at the anoxic boundary within marine sub-surface sediments (in contrast to other benthic species)^31^. Assuming that *C. kullenbergi* δ^13^C reflects bottom-water δ^13^C (ref. 32), the offset of *G. affinis* δ^13^C from bottom water (that is, *C. kullenbergi*) δ^13^C thus sensitively records the relative depletion of pore-water δ^13^C due to organic carbon respiraton^16^,^17^,^30^ driven by the availability of oxygen in bottom waters. The occurrence of *G. affinis* in marine sediments may be in itself an indicator of an oxygen-limited sediment regime, where organic carbon is generally abundant and where the availability of oxygen is the main driver of organic matter respiration within the sediment^29^, because the characteristic zero-oxygen boundary in the shallow sub-surface of these sediments is the preferred habitat of *G. affinis*. The amount of pore water (that is, *G. affinis*) δ^13^C depletion relative to bottom water (that is, *C. kullenbergi*) δ^13^C is thus mostly insensitive to variations in organic carbon fluxes and scales instead with the amount of oxygen diffusing from the bottom water, allowing a quantification of bottom-water [O_2_] (refs 16, 17).

In sediment core MD07-3076Q, *G. affinis* δ^13^C becomes markedly depleted by up to 1‰ relative to bottom-water (*C. kullenbergi*) δ^13^C during decreases in U/Mn_c_ (Fig. 2). The distinct negative offsets of *G. affinis* δ^13^C from *C. kullenbergi* δ^13^C mark millennial-scale increases in deep-water [O_2_] (ref. 16) in the deep sub-Antarctic Atlantic that closely track rises in CO_2,atm_ during the last deglacial and glacial periods (Fig. 2).

According to the modern Δδ^13^C-[O_2_] calibration of ref. 16, bottom-water [O_2_] in the deep sub-Antarctic Atlantic would have reached a minimum of about 40±20 μmol kg^−1^ during the peak glacial, which translates into a bottom-water [O_2_] reduction of 175±20 μmol kg^−1^ from present-day levels of ∼215 μmol kg^−1^ at the core site^33^ (Fig. 2). During the last glacial period, that is, Marine Isotope Stage (MIS) 3, deep sub-Antarctic Atlantic [O_2_] would have varied between 90±25 and 200±40 μmol kg^−1^, in time with millennial-scale changes in CO_2,atm_ (Fig. 2).

Our quantification of deep sub-Antarctic Atlantic [O_2_] relies on the assumption that bottom-water δ^13^C is reliably reflected in *C. kullenbergi* δ^13^C. This species has mostly been employed to reconstruct bottom-water δ^13^C in the southern high latitudes (because of the low abundance of other benthic epifaunal species); yet a difference of up to ∼0.6 ‰ has been observed between sparse glacial *C. kullenbergi* δ^13^C- and glacial *C. wuellerstorfi* δ^13^C measurements at ODP site 1090 in the Cape Basin^34^. This may imply that *C. kullenbergi* δ^13^C is anomalously depleted, for example, due to a slight infaunal habitat during glacial times^34^, and/or that δ^13^C measured on episodically occurring *C. wuellerstorfi* is anomalously enriched, for example, due to an affinity to anomalously well-ventilated water masses^35^ and/or low carbon fluxes. If *C. kullenbergi* δ^13^C in MD07-3076Q does not adequately represent bottom-water δ^13^C at our core site, then absolute bottom-water [O_2_] in the deep central sub-Antarctic Atlantic would be higher by up to ∼40 μmol kg^−1^ per 0.3‰-deviation of glacial bottom-water δ^13^C from glacial *C. kullenbergi* δ^13^C observed in MD07-3076Q (Supplementary Fig. 3). However, our *C. kullenbergi* δ^13^C data are consistent with glacial benthic foraminifer (*C. kullenbergi* and *Cibicidoides* spp.) δ^13^C measurements from different locations throughout the South Atlantic^34^,^36^,^37^, suggesting that they are representative of deep-water δ^13^C. Regardless of these quantitative uncertainties, the co-variation of the U/Mn_c_- and Δδ^13^C-based [O_2_] reconstructions provides strong evidence for recurrent changes in deep sub-Antarctic oxygenation in parallel with CO_2,atm_ over the last glacial and deglacial periods.

### Changes in opal- and organic carbon fluxes

The flux of biogenic silica (opal) to marine sediments in the southern high latitudes is assumed to reflect changes in organic carbon flux to the sea floor and in the export of organic carbon from the euphotic zone (that is, export production)^9^,^38^. Variations in the weight percentages of opal observed in MD07-3076Q are tightly correlated with ^230^Th-normalized opal fluxes (*R*^2^=0.94, *P*<0.05; Fig. 2; Supplementary Fig. 4), suggesting their accurate representation of past opal- (and therefore total organic carbon^9^,^38^; TOC) fluxes in the sub-Antarctic Atlantic. This is supported by synchronous variations in the TOC flux observed in the neighbouring core PS2498-1 (Fig. 2, location in Fig. 1), which has been chronostratigraphically aligned to MD07-3076Q (Methods). As shown in Fig. 2, opal- and TOC fluxes in the sub-Antarctic Atlantic show a close link to dust flux variations in Antarctic ice cores and changes in dust supply to the sub-Antarctic region^9^, which is consistent with earlier findings^9^,^10^.

### Estimates of radiocarbon ventilation ages

**Figure 3:**
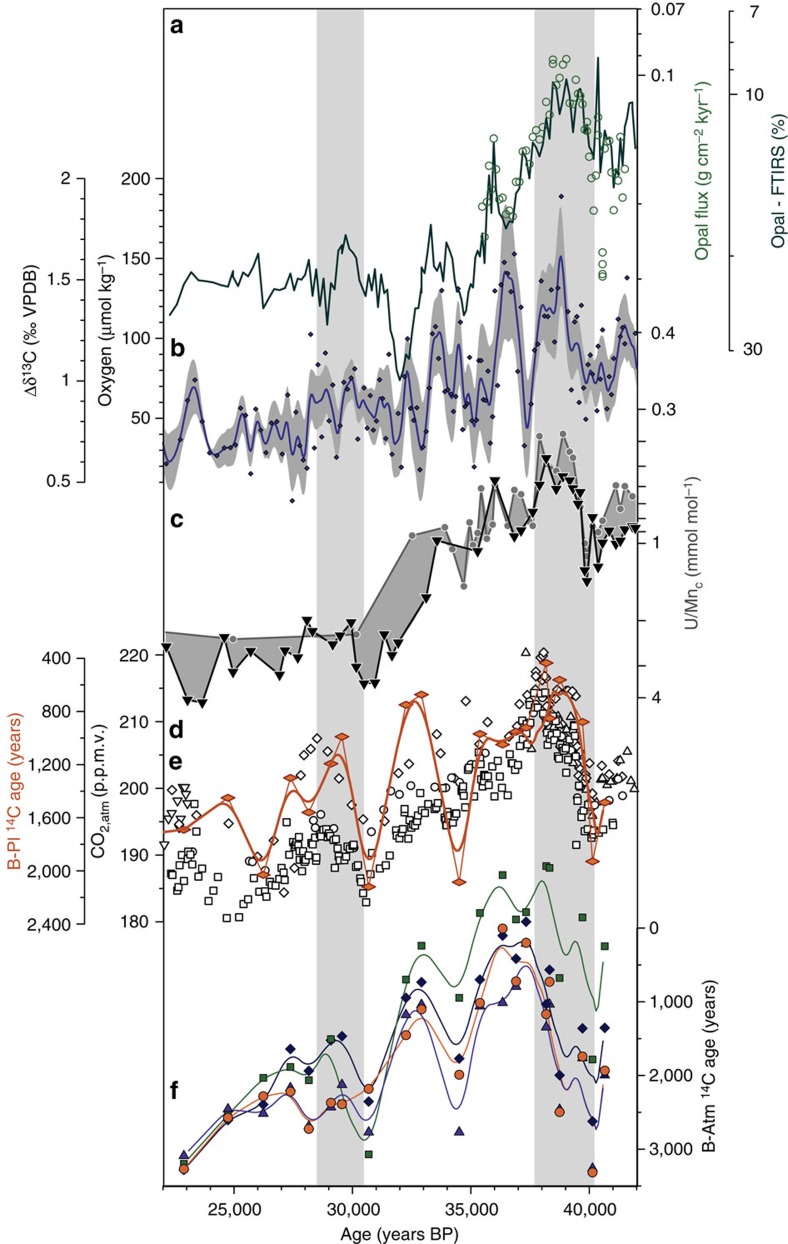
Mid-glacial ventilation and carbon sequestration changes in the deep sub-Antarctic Atlantic. (**a**) Sedimentary opal content (line) and ^230^Thorium-normalized opal fluxes (circles), (**b**) Δδ^13^C_*C. kullenbergi*–*G. affinis*_ and corresponding bottom-water [O_2_] (ref. 16), (**c**) *G. bulloides* (circles) and *Uvigerina* spp. (triangles) U/Mn_c_, (**d**) Benthic-Planktonic (B-Pl) ^14^C ventilation ages and the corresponding 1,000 years-running mean (thick line) plotted on top of (**e**) variations in CO_2,atm_ recorded in the Antarctic ice cores (open symbols, refs as in Fig. 2), (**f**) benthic foraminifer ^14^C age offset from atmospheric ^14^C (Lake Suigetsu (green)^74^, Cariaco Basin (orange)^75^, Intcal09 (blue)^76^ and Intcal13 (dark blue)^77^) shown as 1,000 years-running means (lines). Line and grey envelope in **b** show a 500 year-running average and the 500 year-window one-sigma standard deviation, respectively. Grey bars indicate periods of rising CO_2,atm_.

Two metrics for deep-water ‘ventilation' (that is, deep ocean versus atmosphere gas/isotope equilibration) that provide a measure of the average time since carbon in the ocean interior last equilibrated with the atmosphere are considered here: ^14^C age offsets between coexisting benthic (B) and planktonic (Pl) foraminifera (B-Pl ^14^C ventilation ages), and benthic ^14^C age offsets from contemporary atmospheric ^14^C ages (B-Atm ^14^C ventilation ages). While the first provide an estimate of deep-ocean ventilation relative to the local mixed layer, the latter provide a direct estimate of deep-ocean ventilation relative to the contemporary atmosphere. As shown in Fig. 3, B-Pl ^14^C ventilation ages from sediment core MD07-3076Q broadly co-vary with changes in deep-ocean oxygenation (for example, with U/Mn_c_: *R*^2^=0.31, *P*<0.05) and CO_2,atm_ (*R*^2^=0.43, *P*<0.05), both statistically significant within the 95% significance interval (Supplementary Fig. 5). Parallel B-Atm ^14^C ventilation age estimates agree with these observations, and confirm that B-Pl ^14^C ventilation age fluctuations have not been significantly biased or masked by local surface-ocean radiocarbon disequilibrium effects (reservoir ages) (Fig. 3).

These findings are consistent with similar analyses in the central deep sub-Antarctic Atlantic for the last deglaciation^14^. Although B-Pl ^14^C ventilation age variations are more strongly influenced by surface-ocean reservoir age variations during the last deglaciation, decreasing B-Atm ^14^C age offsets are linked to deglacial increases in CO_2,atm_, in particular during the early deglacial period^14^.

Notably, absolute foraminifer ^14^C ages appear to be slightly too young during the mid-glacial period, perhaps due to uncertainties associated with background corrections, which are especially important for old (>30 kyr BP) sample material. In practice, these background corrections are based on one radiocarbon-dead spar calcite sample measured in each sample batch (that is, an accelerator mass spectrometry (AMS) sample carousel), whose apparent radiocarbon content is subtracted from the measured radiocarbon content of all the foraminifer samples measured in that sample carousel. If the true background deviates from the measured background in this single sample, then B-Atm ^14^C and Pl-Atm ^14^C age offsets may deviate significantly from their true absolute values. Godwin Radiocarbon Laboratory-internal backgrounds compiled for the 4 years from April 2011 to January 2015 amount to ^14^C/^12^C_0_=5.3±1.5 × 10^−15^ (Supplementary Fig. 6). Considering a one-off estimate of the background that is slightly smaller (that is, ^14^C/^12^C_0_=4 × 10^−15^; within 1 s.d. of the mean) or larger (that is, ^14^C/^12^C_0_=6 × 10^−15^; within 1 s.d. of the mean), this would result in B-Atm ^14^C and Pl-Atm ^14^C age offsets that are shifted towards slightly lower and higher absolute values respectively, without affecting the overall variability in each time-series (Supplementary Fig. 7). As benthic and planktonic ^14^C ages have been obtained from the same AMS sample carousels in this study, B-Pl ^14^C ventilation ages are not affected by these uncertainties and are essentially the same irrespective of the applied background correction (Supplementary Fig. 7). Therefore, while our absolute B-Atm ^14^C and Pl-Atm ^14^C age offsets are dependent on the accuracy of our background corrections (which are arguably difficult to assess), relative changes in B-Atm ^14^C ventilation ages and absolute variations in B-Pl ^14^C ventilation ages remain robust. As shown in Fig. 3, these clearly co-vary with our estimates of bottom-water oxygenation in the deep sub-Antarctic Atlantic (see also Supplementary Fig. 5).

## Discussion

Changes in the elemental composition of foraminifer coatings and bottom-water versus pore-water δ^13^C gradients, as described above, demonstrate that the amount of remineralized carbon sequestered in the deep sub-Antarctic Atlantic has varied substantially and inversely with respect to millennial-scale CO_2,atm_ changes (Fig. 2). These observations confirm a role for the Southern Ocean ‘organic carbon pump' in regulating CO_2,atm_ (refs 3, 10, 11). Below, we assess the quantitative impact of the inferred ‘biological carbon pump' changes on CO_2,atm_, as well as their governing biological and/or physical/dynamical controls.

Bottom-water [O_2_] reconstructions at our core site via Δδ^13^C_*C. kullenbergi*–*G. affinis*_ provide the basis for a quantification of the amount of respired carbon in the deep sub-Antarctic Atlantic^16^,^30^, provided the modern Δδ^13^C-[O_2_] relationship holds for the past. In principle, seawater [O_2_] consists of a saturated [O_2_] component ([O_2_]_sat_) arising from the solubility-controlled O_2_ exchange between the atmosphere and the surface ocean, a biological [O_2_] component associated with the release and drawdown of [O_2_] during photosynthesis and respiration ([O_2_]_bio_), and a preformed disequilibrium [O_2_] component ([O_2_]_diseq_) due to inefficiencies in air–sea gas exchange ([O_2_]_*insitu*_=[O_2_]_sat_+[O_2_]_bio_+[O_2_]_diseq_)^39^. Assuming that ocean [O_2_] is in equilibrium with the atmosphere ([O_2_]_diseq_∼0) and that last glacial ocean [O_2_]_sat_ was slightly higher than today ([O_2_]_sat,modern_=345 μmol kg^−1^) mostly due to a decrease in ocean temperature ([O_2_]_sat,glacial_=360 μmol kg^−1^; Methods), the amount of [O_2_] depletion at ocean depth (AOU=−[O_2_]_bio_=[O_2_]_sat_−[O_2_]_*insitu*_) should scale with the formation of respired carbon according to a constant stoichiometric Redfield ratio of C:[O_2_]=117:−170 (ref. 40). Changes in bottom-water [O_2_] (and AOU) in the sub-Antarctic Atlantic would therefore provide a direct quantitative measure of the amount of carbon sequestered in the southern high-latitude ocean, and thus the efficiency of the biological ‘organic carbon pump'^16^.

Converting our AOU estimates into respired carbon concentrations (AOU_Holocene_=(345−215) μmol kg^−1^, (AOU_MIS2_=(360−40)±20 μmol kg^−1^, ΔAOU_Deglaciation_=190±20 μmol kg^−1^)based on the Redfield ratio of C:[O_2_]=117:−170 (ref. 40) gives a respired DIC contribution of 220±14 μmol kg^−1^ to the total DIC pool at the core site during the last glacial maximum (LGM). This is higher by 130±14 μmol kg^−1^ compared with the Holocene^33^, indicating greater respired carbon accumulation during the LGM. During millennial-scale variations in CO_2,atm_ during the last glacial, respired carbon levels varied by 75±28 μmol kg^−1^ between 110±28 μmol kg^−1^ (during peak CO_2,atm_ levels) and 185±14 μmol kg^−1^ (during minimum CO_2,atm_ levels) assuming that [O_2_]_sat_ was not significantly different from LGM levels (that is, AOU_MIS3‘CO_2_max'_=(360−200)±40 μmol kg^−1^, AOU_MIS3‘CO_2_min'_=(360−90)±25 μmol kg^−1^, ΔAOU_MIS3_=110±40 μmol kg^−1^).

If we assume that the respired carbon lost from the deep sub-Antarctic Atlantic, where it was sequestered away from the atmosphere was transferred to a non-respired marine carbon pool that in turn equilibrated with the atmosphere via a surface-ocean DIC ‘buffer factor' (that is, Revelle factor) of ∼10 (ref. 41; Methods), our AOU and respired carbon estimates from the deep sub-Antarctic may only explain the full amplitude of observed CO_2,atm_, if they are representative of a significant fraction of the global deep ocean, that is, at least ∼33% during the mid-glacial period and ∼45% during the early deglaciation (Methods). This would roughly correspond to the deep ocean below 2.9 and 2.3 km, respectively (Methods). These depths broadly agree with the depth of the putative glacial ‘chemical divide' (∼3 km water depth in the Atlantic)^42^, and are supported by qualitative proxy data showing a decrease in oxygenation and radiocarbon ventilation in the global ocean below 2 km during the last peak glacial period^43^,^44^. The smaller the volume of the global deep ocean that experienced similar changes in AOU and respired carbon to our sub-Antarctic Atlantic site, the smaller the likely oceanic impact on CO_2,atm_ concentrations.

Our calculations have two major caveats. First, we ignore possible open-system effects due to the interaction of deep waters with sediments, and second, we may have underestimated glacial deep sub-Antarctic Atlantic [O_2_], in the case that deep sub-Antarctic Atlantic *C. kullenbergi* δ^13^C values are strongly negatively biased versus bottom-water δ^13^C. Any open-system effects involving a degree of ‘carbonate compensation' (on multi-millennial timescales) would tend to enhance the impact of marine respired carbon inventory changes on CO_2,atm_ (Methods). If true glacial bottom-water [O_2_] were higher than estimated in MD07-3076Q for instance via an anomalous depletion of *C. kullenbergi* δ^13^C from bottom-water δ^13^C by 0.3‰, as mentioned above, deglacial changes in respired carbon and AOU (and thus the oceanic impact on CO_2,atm_) would be reduced by ∼20%, as LGM AOU values would be lower (Supplementary Fig. 3). In contrast, our estimates of respired carbon changes during the last mid-glacial period remain to a large extent similar as they are based on relative [O_2_] changes (Supplementary Fig. 3). Our calculations are rough estimates that are intended only to provide a first indication of the potential impact of our observed marine carbon sequestration changes on CO_2,atm_. To determine the full impact of changes in deep-ocean respired carbon levels on CO_2,atm_ concentrations, our estimates would need to be corroborated by further reconstructions of past bottom-water oxygen- and DIC concentrations from throughout the global ocean, in particular in the volumetrically most significant Pacific Ocean.

The analysis above demonstrates the potential quantitative significance of the oxygenation changes that we observe, and more specifically of the role of the Southern Ocean ‘organic carbon pump' in regulating CO_2,atm_ (refs 3, 10, 11). However, it remains to be shown whether the observed decreases in ‘organic carbon pump' efficiency resulted primarily from decreases in export productivity (allowing oxygen to increase due to reduced organic carbon remineralization in the ocean interior) or primarily from increases in ocean ‘ventilation' (causing carbon loss to the atmosphere with direct oxygen gain of the ocean interior). Below, we address this question by reference to our export productivity- and ^14^C ventilation age estimates.

The observed correlation between changes in the dust supply to the southern high-latitude regions and in export production in the central sub-Antarctic Atlantic (as recorded by variations in opal- and TOC fluxes^9^; Fig. 2) supports earlier findings of a dust-driven biological organic carbon pump in the sub-Antarctic Atlantic^9^,^10^. The close relationship between variations in sub-Antarctic export production and CO_2,atm_ changes (Fig. 2) would be consistent with a significant impact of the efficiency of the sub-Antarctic biological organic carbon pump on surface-ocean DIC levels, and thus on CO_2,atm_ (refs 9, 10, 11).

The correlation between opal- and TOC fluxes and bottom-water [O_2_] in the sub-Antarctic Atlantic (Fig. 2) may point to a role of organic carbon respiration at depth driving deep sub-Antarctic Atlantic bottom-water oxygenation. To test whether export production was the major driver of our observed deep-ocean [O_2_] changes (and therefore of the associated changes in deep-ocean respired carbon sequestration), we make use of the unique microhabitat of *G. affinis* near the anoxic boundary in marine sediments and the associated mechanisms that drive its δ^13^C signature. Notably, negative excursions of *G. affinis* δ^13^C are observed during each of the marked CO_2,atm_ rises during MIS 3 and the last deglaciation. These excursions indicate that total organic carbon respiration within deep sub-Antarctic Atlantic sediments increased at times of reduced opal- and TOC fluxes, that is, reduced export production (Fig. 2). An increase in sedimentary organic carbon respiration (that is, pore water/*G. affinis* δ^13^C depletions) would be driven by an increase in organic carbon flux, an increase in bottom-water [O_2_], or both of these together. As the first is evidently not the case (Fig. 2), we conclude that sedimentary carbon respiration must have instead been driven by enhanced deep-ocean ‘ventilation' (that is, circulation/ convection rates and/or air–sea gas exchange) supplying oxygen to the deep sub-Antarctic Atlantic.

Alternatively, a decreased oxygen demand in bottom waters due to diminished organic carbon fluxes and less respiration of organic matter in a benthic ‘fluff' layer could facilitate the diffusion of oxygen into the sediment, and drive the *G. affinis* δ^13^C signal more negative. However, a poor inverse correlation between epibenthic and deep infaunal benthic foraminifer δ^13^C over past millennial timescales (Fig. 2; *R*^2^=0.0001, *N*=258, Supplementary Fig. 8) would appear to rule out this scenario. We therefore conclude that the observed changes in ‘organic carbon pump' efficiency and deep sub-Antarctic carbon storage were not only controlled by changes in export productivity but must also have involved biology-independent processes that contributed to past CO_2,atm_ changes specifically by enhancing ocean–atmosphere CO_2_ exchange in the Antarctic region (Fig. 2).

Our interpretation is confirmed by parallel estimates of deep-water ^14^C ‘ventilation ages' (Fig. 3). We observe that the marked CO_2,atm_ rise around 38 kyr BP is paralleled by a decrease in B-Atm ^14^C ventilation ages of ∼2,000 ^14^C years. A consistent link between deep-ocean (B-Atm and B-Pl) ^14^C ventilation and CO_2,atm_ variability is further supported by a high and statistically significant correlation coefficient between them (up to *R*^2^=0.6, *P*<0.05; Supplementary Fig. 5). The good correlation between (B-Atm and B-Pl) ^14^C ventilation ages, deep-water [O_2_] and CO_2,atm_ provides strong independent support for changes in the air–sea equilibration of deep waters in the Southern Ocean and their link to changes in respired carbon storage.

**Figure 4:**
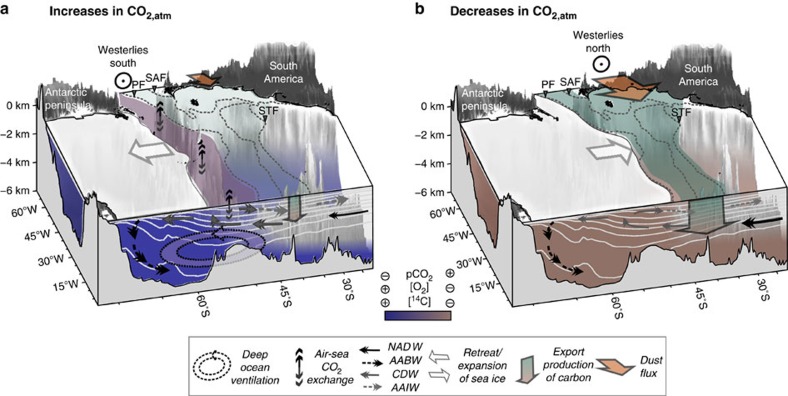
Schematic view on the southern high-latitude Atlantic during millennial-scale CO_2,atm_ variations based on new and existing proxy evidence. (**a**) Dust-driven decreases of export production in the sub-Antarctic Atlantic^9^,^10^ during the last glacial and deglacial periods were accompanied by decreases in deep carbon storage in the Southern Ocean (this study and ref. 48). The latter was further promoted by increases in the air-sea CO_2_ exchange south of the PF and in the ventilation of the deep carbon pool (this study and ref. 48), causing millennial-scale increases in CO_2,atm_, as postulated earlier^3^,^7^. (**b**) Enhanced dust-driven, biological export of carbon to the deep sub-Antarctic Atlantic^9^,^10^ paralleled increases in deep Southern Ocean respired carbon levels during the last glacial period and the last deglaciation (this study and ref. 48). The enhanced Southern Ocean carbon pool was effectively isolated from the atmosphere by decreases in air–sea CO_2_ equilibration in the Antarctic region and a poor 'ventilation' of the deep-ocean during these times (this study and ref. 48), leading to decreases in CO_2,atm_ during the last 70,000 years, as proposed previously^3^,^7^. Accompanying changes in sea ice^5^,^6^ and the westerly position/strength^7^,^8^ are debated and remain speculative. The modern positions of ocean fronts (as in Fig. 1) and ocean density surfaces (white lines) are shown as reference.

It has previously been shown that the incursion of well-ventilated northern-sourced waters into the sub-Antarctic Atlantic was reduced during intervals of rising CO_2,atm_ (refs 20, 28). On this basis, the periods of increased ^14^C ventilation that we observe would therefore specifically reflect periods of increased local dominance of southern-sourced deep waters and an ‘improvement' of their ventilation state. Numerous processes have been suggested to have caused changes in vertical mixing in the southern high latitudes, including for instance the intensity and/or the position of the southern hemisphere westerlies^7^,^8^, a retreat of circum-Antarctic sea ice^6^, a decline in the formation and advection of northern component waters^45^ and/or changes in surface buoyancy fluxes^46^. It remains currently impossible to evaluate the relative importance of these specific processes and their controls on CO_2,atm_. Nevertheless, the strong co-variations of our abyssal oxygenation and ventilation proxies with CO_2,atm_ confirm that some combination of dynamical (that is, residual circulation and shallow mixing) and/or physical (gas exchange efficiency) processes in the southern high-latitude region indeed had a significant impact on deep-ocean carbon sequestration^3^,^7^,^19^,^45^,^47^ (Fig. 4).

Furthermore, our findings are entirely consistent with recently published sedimentary redox-sensitive trace element data from the Antarctic Zone of the Atlantic Ocean^48^. These data show that the accumulation of authigenic uranium (and therefore oxygenation) in the Antarctic Atlantic is generally inversely correlated with opal fluxes (that is, organic carbon fluxes) over the past 80,000 years, ruling out a dominant control of local surface-ocean productivity on deep Antarctic Atlantic [O_2_] and deep-ocean respired carbon levels south of the PF (ref. 48). The combination of our sub-Antarctic study with the Antarctic study of ref. 48 provides strong evidence for millennial-scale changes in the respired carbon concentrations across the entire deep high-latitude South Atlantic, varying in parallel with CO_2,atm_ during the last glacial period and deglaciation, and for a significant impact of physical ‘ventilation' processes (that is, overturning circulation, mixing and/or air–sea gas exchange) on changes in deep-ocean respired carbon sequestration and millennial-scale CO_2,atm_ in the past.

In conclusion, our results show that pulses of CO_2,atm_ during the last glacial- and deglacial periods coincided with increases in the ventilation of the southern high-latitude deep ocean (specifically via regions of deep-water formation in the Southern Ocean^7^,^48^), in addition to reductions in sub-Antarctic export productivity. By ruling in a role for variations in both the strength and the efficiency of the biological carbon pump via changes in the biological carbon export as well as the air–sea CO_2_ exchange and Southern Ocean vertical mixing, the findings reconcile two opposing theories for the Southern Ocean's role in past millennial-scale CO_2,atm_ variability^3^,^7^,^10^,^11^,^12^,^47^. Further work, for example using numerical model simulations will be required to quantify more precisely the contributions of (sub-polar zone) biological export productivity changes and (polar zone) physical/dynamical changes to deep-ocean carbon sequestration, as well as their down-stream effects on low-latitude export production^49^. Nevertheless, our data emphasize that while biological carbon export to the deep ocean is ultimately what permits ocean dynamics and air–sea exchange to impact on CO_2,atm_ by continually tending to ‘recharge' the abyssal carbon pool, the rate of equilibration of the deep ocean with the atmosphere will ultimately determine whether or not the biological ‘organic carbon pump' is efficient or not at sequestering CO_2_ (Fig. 4). Thus, ocean physics and marine biology acted together, synergistically, to repeatedly nudge the Southern Ocean from carbon sink to carbon source, with a direct impact on global climate over the last ∼65,000 years.

## Methods

### Regional setting and chronology

Sediment core MD07-3076Q (14°13.7′W, 44°9.2′S, 3,770 m water depth) is bathed in Lower Circumpolar Deep Water, which is formed by the entrainment of northward spreading DIC- and preformed nutrient-rich Circumpolar Deep Water into southward flowing DIC-low and regenerated nutrient-rich North Atlantic Deep Water^50^. Chronological control of sediment core MD07-3076Q is based on ^14^C measurements of mono-specific planktonic foraminifer samples, which have been adjusted for variations in surface-ocean reservoir ages^14^. The ^14^C-based age constraints are complemented by the stratigraphic alignment of abundance variations of the cold-water species *Neogloboquadrina pachyderma* (sinistral-coiling) with rate changes in Antarctic temperature over time^19^. Age model uncertainties, mainly a function of age marker density, amount to 1,600±500 years during the last glacial period and to 1,200±400 years after 27 kyr BP (ref. 19). Resulting sedimentation rates range between 5 cm kyr^−1^ during the last deglaciation and 15 cm kyr^−1^ during MIS 3.

### Element composition of authigenic foraminifer coatings

Down-core measurements of U/Ca_cc+c_ and U/Mn_c_ have been made on 18–25 specimens of the planktonic foraminifer *G. bulloides* (250-300 μm size fraction) and the 5–13 specimens of the benthic infaunal foraminifer *Uvigerina* spp. (250-300 μm size fraction). Foraminifera have been weakly chemically cleaned (clay removal and silicate picking) to maintain foraminiferal coatings but to remove extraneous detritus^15^. Cleaned foraminifera have been dissolved in 0.1 M nitric acid for inductively coupled plasma (ICP)-atomic emission spectroscopy analyses. The samples were subsequently re-diluted to 10 p.p.m. Ca^2+^ concentration and elemental concentrations have been determined by ICP-mass spectrometry^15^. Mean s.d. of U/Mn_c_ of six duplicate samples is 0.08±0.06 mmol mol^−1^. Given the high sedimentation rates of 15 cm kyr^−1^, the impact of potential sedimentary re-oxidation processes (‘burn-down' effects) of already precipitated uranium complexes is negligible for the interpretation of U/Ca_cc+c_ and U/Mn_c_ ratios.

### Reconstruction of bottom- to pore-water δ^13^C gradients

Stable isotopic analyses on *G. affinis* and *C. kullenbergi* have been performed on 1–4 specimens (>150 μm size fraction) on Finnigan Δ+ and Elementar Isoprime mass spectrometers. The results are reported with reference to the international Vienna Pee Dee Belemnite (VPDB) standard. VPDB is defined with respect to the NBS-19 calcite standard. The mean external reproducibility of carbonate standards is σ±0.03 ‰.

In MD07-3076Q, Δδ^13^C_*C. kullenbergi–G. affinis*_ has been determined from δ^13^C measurements of benthic foraminifera from the same sediment sample, and has been converted into bottom-water [O_2_] after ref. 16. The calibration error associated with bottom-water [O_2_] reconstructions using this method is ±17 μmol kg^−1^ (ref. 16). Analytical uncertainties of benthic δ^13^C analyses (two-sigma) translate into a bottom-water [O_2_] uncertainty of ±8 μmol kg^−1^. We have smoothed our high-resolution record by a running 500 year-window (solid line in Fig. 2) to reduce such biases and those from intra-specific δ^13^C variations. Mean bottom-water [O_2_] have been determined for the LGM (23-18 kyr BP) as well as CO_2_ minima (40.2-39.9 kyr BP, 48.4-47.6 kyr BP, 56.7-55.7 kyr BP, 63.6-63.0 kyr BP) and -maxima (38.8-38.0 kyr BP, 46.3-45.8 kyr BP, 53.6-53.3 kyr BP and 59.3-58.8 kyr BP) during MIS 3. Errors reported in our study are one-sigma standard deviations of our bottom-water [O_2_] estimates during these periods.

### Calculation of deep-ocean and atmospheric carbon budgets

[O_2_] saturation levels are calculated according to ref. 51 assuming a glacial increase in salinity from present-day (∼35 p.s.u.) by ∼2 p.s.u. and a decrease in deep-ocean temperatures from modern-day values (∼1 °C) by 2 °C in the deep Southern Ocean^52^. [O_2_] saturation in the glacial deep Southern Ocean increased by ∼15 μmol kg^−1^ from modern-day levels (∼345 μmol kg^−1^) (ref. 33).

To estimate the amount of carbon that is transferred to the atmosphere from the ocean's remineralized carbon pool (sequestered in the deep ocean), via the ocean's non-remineralized carbon pool (in equilibrium with the atmosphere), we adopt the conceptual framework of ref. 41, whereby:


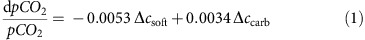


Here, Δ*c*_soft_ and Δ*c*_carb_ are DIC changes for the ocean's total remineralized carbon pool (that is not in equilibrium with the atmosphere), due to changes in the soft-tissue pump and the carbonate pump (for instance via changes in the export of organic carbon and carbonate to the ocean interior), respectively. Our estimate of Δ*c*_DIC_ during the last deglacial increase in CO_2,atm_ (Δ*c*_DIC_=130±14 μmol kg^−1^) and during mid-glacial CO_2,atm_ changes (Δ*c*_DIC_=75±28 μmol kg^−1^) determined above from oxygenation estimates provides an estimate of Δ*c*_soft_, during these time intervals, and we assume that the associated Δ*c*_soft_ is approximately three times smaller (for example, as observed spatially in the modern ocean)^41^, yielding:


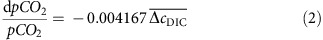


where 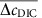 is the whole-ocean average change in remineralized carbon during the investigated time intervals. It is given by the product of the change observed at our core location and the fraction (*f*) of the total ocean volume that also experienced this magnitude of change:


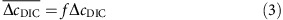


Assuming that the rest of the ocean volume experienced no significant change in respired DIC, remaining well-equilibrated with the atmosphere, the fraction of the ocean *f*, and therefore the deep-ocean volume *V*_d_ and the upper ‘boundary' of the deep-ocean *z'*, may be calculated that would account for the last early deglacial and mid-glacial atmospheric pCO_2_ changes of ∼50 and ∼20 p.p.m. (for glacial background pCO_2_ levels of 190–200 p.p.m.), if affected by similar changes in AOU and respired DIC levels as our sub-Antarctic Atlantic core site.

We have calculated the deep-ocean volume *V*_d_ and *z'* based on the GEBCO bathymetric data set (excluding the Arctic Ocean) archived by the British Oceanographic Data Centre (http://www.gebco.net/), according to:


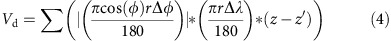


that is the sum of all volumes of grid boxes (distance in west-east direction (km) times distance north-south direction (km) times depth), where *φ* is latitude, *λ* is longitude, Δ*φ* and Δ*λ* represent the grid spacing of the bathymetric data set, *r* is the Earth's radius, *z* the water depth and *z'* the upper limit of the deep ocean.

### Opal measurements

Opal concentrations were measured on ∼400 samples by means of Fourier transform infrared (FTIR) spectroscopy^53^ using a Vertex 70 FTIR-spectrometer (Bruker Optics Inc.) at the Institute of Geological Sciences at the University of Bern (CH). The FTIR spectra have been independently calibrated based on FTIRS analyses of artificial sand/opal mixtures^54^. Opal concentrations determined by means of FTIR spectroscopy show excellent agreement with conventional photometric-based^55^ opal concentration determinations (*R*^2^=0.91; Supplementary Fig. 4) that have been performed on one quarter of the total number of samples (*N*=101). However, an increasing offset between photometric and FTIR-based opal measurements towards increasing opal values (Supplementary Fig. 4) might point at incomplete alkaline opal dilution during photometric measurements^55^, potentially caused by a significant fraction of radiolarian skeletons in MD07-3076Q sediments^56^.

Opal fluxes have been determined by normalizing the opal data with measured ^230^Th concentrations^57^. For these analyses, U- and Th- isotopes were analysed by means of ICP–quadrupole mass spectrometry (iCAP-Q ICP-MS, ThermoFisher) at the Institute for Environmental Physics in Heidelberg, Germany. The contribution of detrital ^230^Th has been estimated by assuming a ^238^U/^232^Th ratio of 0.6 and a correction^58^ for the detrital ^234^U/^238^U not in secular equilibrium of 0.96. The quality of the analyses and the sample digestion and purification process has been monitored by blanks, certified UREM-11 standard material and replicate measurements of samples. Full replicates (*N*=5) yielded an average uncertainty of 2.8 % (two-sigma) of the excess ^230^Th concentrations (Supplementary Table 1). The chosen parameter set for the measurements of marine sediments applied here for the first time using an iCAP-Q ICP-MS (Supplementary Tables 1 and 2) puts emphasis on time efficiency for high-matrix sample analyses.

### Radiocarbon measurements

The previously published set of foraminiferal ^14^C dates in sediment core MD07-3076Q (ref. 14) has been extended by additional paired ^14^C measurements of mixed benthic and mono-specific planktonic foraminifera (*N. pachyderma* s.). The conventional ^14^C ages are reported in Supplementary Tables 3 and 4. The mean ^14^C age uncertainty of the new ^14^C data set amounts to 650±270 ^14^C years (Supplementary Table 3).

Foraminifer samples had a mean weight of 5.1±1.0 mg, and weighed always more than 3.4 mg. They have been gently cleaned in methanol, and were subsequently transferred to sealed septum vials after they were completely dry. After evacuation 0.5 ml dry phosphoric acid has been injected into the vials. The acid-carbonate reaction has been sustained for at least 0.5 h at 60 °C. The CO_2_ samples were graphitized in the Godwin Radiocarbon Laboratory at the University of Cambridge (UK), along with standards and radiocarbon-dead spar calcite (backgrounds), following a standard hydrogen/iron catalyst protocol^59^. Pressed graphite targets were subsequently analysed by AMS at the ^14^Chrono Centre, University of Belfast (UK). Measured ^14^C ages have been corrected for mass-dependent fractionation (normalization to δ^13^C=−25‰) and the background radiocarbon content by analysing radiocarbon-dead spar calcite with each sample batch. Paired planktonic and benthic samples have been measured in the same AMS sample carousel.

Four paired measurements have resulted in younger benthic than planktonic foraminifera (Supplementary Fig. 5). We have omitted these data from the initial analyses, but including these samples does not alter the general trend of the data (Supplementary Fig. 5).

### Correlation of marine proxy records with CO_2,atm_ variations

To calculate correlation coefficients *R*^*2*^ between CO_2,atm_ variations and ^14^C-based deep sub-Antarctic ventilation ages during the last glacial period, that is, 41-22 kyr BP (Supplementary Fig. 5e), we interpolated the mean CO_2,atm_ record^19^ at the sampling resolution of the ^14^C proxy data. Similarly, the mean CO_2,atm_ has been interpolated at the resolution of the mean U/Mn_c_- and the Δδ^13^C-based [O_2_] records in order to estimate the correlation (*R*^*2*^) between changes in bottom-water oxygenation and ^14^C ventilation in the deep sub-Antarctic Atlantic (Supplementary Fig. 5f,g). For these calculations, the mean U/Mn_c_ has been obtained by averaging *G. bulloides* and *Uvigerina* spp. U/Mn_c_ (stippled line in Supplementary Fig. 2a) and the Δδ^13^C-derived [O_2_] record is based on a 500 year-running average (solid line in Fig. 2f).

### Chronostratigraphy of other sub-Antarctic Atlantic cores

The most recent age model of sediment core PS2498-1 has been established based on an alignment of variations in lithogenic fluxes with the EPICA Dome C dust record^9^. Because sediment cores MD07-3076Q and PS2498-1 are in close proximity (Fig. 1), we have compared the magnetic susceptibility records and noticed stratigraphic offsets of ±900 years. To allow a faithful inter-core comparison, we have adjusted the chronology of PS2498-1 by aligning the magnetic susceptibility record of PS2498-1 (ref. 60) to the magnetic susceptibility record of MD07-3076Q, which has been measured with the GEOTEK Multi-Sensor-Core-Logger aboard *R/V Marion Dufresne* using a low field susceptibility (Bartington) sensor. For TN057-21, we rely on the most recently established chronology of ref. 28, which is based on the GICC05 age scale^61^ that is equivalent to the AICC2012 age scale used in this study within decades to few hundred years^62^.

## Additional information

**How to cite this article:** Gottschalk, J. *et al*. Biological and physical controls in the Southern Ocean on past millennial-scale atmospheric CO_2_ changes. *Nat. Commun.* 7:11539 doi: 10.1038/ncomms11539 (2016).

## Supplementary Material

Supplementary Information Supplementary Figures 1-8, Supplementary Tables 1-4 and Supplementary References.

## References

[b1] Sigman D. M. & Boyle E. A. Glacial/interglacial variations in atmospheric carbon dioxide. Nature 407, 859–869 (2000). 11057657 10.1038/35038000

[b2] Ito T. & Follows M. J. Preformed phosphate, soft tissue pump and atmospheric CO_2_. J. Mar. Res. 63, 813–839 (2005).

[b3] Schmittner A. & Galbraith E. D. Glacial greenhouse-gas fluctuations controlled by ocean circulation changes. Nature 456, 373–376 (2008). 19020618 10.1038/nature07531

[b4] Martin J. H. Glacial-interglacial CO_2_ change: the iron hypothesis. Paleoceanography 5, 1–13 (1990).

[b5] Ferrari R. . Antarctic sea ice control on ocean circulation in present and glacial climates. Proc. Natl. Acad. Sci. USA 111, 8753–8758 (2014). 24889624 10.1073/pnas.1323922111 PMC4066517

[b6] Stephens B. B. & Keeling R. F. The influence of Antarctic sea ice on glacial-interglacial CO_2_ variations. Nature 404, 171–174 (2000). 10724166 10.1038/35004556

[b7] Anderson R. F. . Wind-driven upwelling in the Southern Ocean and the deglacial rise in atmospheric CO_2_. Science 323, 1443–1448 (2009). 19286547 10.1126/science.1167441

[b8] Toggweiler J. R., Russell J. L. & Carson S. R. Midlatitude westerlies, atmospheric CO_2_, and climate change during the ice ages. Paleoceanography 21, 2005 (2006).

[b9] Anderson R. F. . Biological response to millennial variability of dust and nutrient supply in the Subantarctic South Atlantic Ocean. Philos. Trans. R. A Math. Phys. Eng. Sci. 372, 20130054 (2014). 10.1098/rsta.2013.0054 24891398

[b10] Martínez-García A. . Iron fertilization of the Subantarctic Ocean during the last ice age. Science 343, 1347–1350 (2014). 24653031 10.1126/science.1246848

[b11] Jaccard S. L. . Two modes of change in Southern Ocean productivity over the past million years. Science 339, 1419–1423 (2013). 23520109 10.1126/science.1227545

[b12] Ziegler M., Diz P., Hall I. R. & Zahn R. Millennial-scale changes in atmospheric CO_2_ levels linked to the Southern Ocean carbon isotope gradient and dust flux. Nat. Geosci. 6, 457–461 (2013).

[b13] Frank M. . Similar glacial and interglacial export bioproductivity in the Atlantic sector of the Southern Ocean: multiproxy evidence and implications for glacial atmospheric CO_2_. Paleoceanography 15, 642–658 (2000).

[b14] Skinner L. C., Fallon S., Waelbroeck C., Michel E. & Barker S. Ventilation of the deep Southern Ocean and deglacial CO_2_ rise. Science 328, 1147–1151 (2010). 20508128 10.1126/science.1183627

[b15] Boiteau R., Greaves M. & Elderfield H. Authigenic uranium in foraminiferal coatings: a proxy for ocean redox chemistry. Paleoceanography 27, PA3227 (2012).

[b16] Hoogakker B. A. A., Elderfield H., Schmiedl G., McCave I. N. & Rickaby R. E. M. Glacial – interglacial changes in bottom-water oxygen content on the Portuguese margin. Nat. Geosci. 8, 40–43 (2015).

[b17] McCorkle D. C., Keigwin L. D., Corliss B. H. & Emerson S. R. The influence of microhabitats on the carbon isotopic composition of deep-sea benthic foraminifera. Paleoceanography 5, 161–185 (1990).

[b18] Anderson R. F. . Biological response to millennial variability of dust supply in the Subantarctic South Atlantic Ocean. Philos. Trans. R. A Math. Phys. Eng. Sci. 372, 20130054 (2014). 10.1098/rsta.2013.0054 24891398

[b19] Gottschalk J., Skinner L. C. & Waelbroeck C. Contribution of seasonal sub-Antarctic surface water variability to millennial-scale changes in atmospheric CO_2_ over the last deglaciation and Marine Isotope Stage 3. Earth Planet. Sci. Lett. 411, 87–99 (2015).

[b20] Gottschalk J. . Abrupt changes in the southern extent of North Atlantic Deep Water during Dansgaard-Oeschger events. Nat. Geosci. 8, 950–955 (2015).

[b21] Russell A. D., Hönisch B., Spero H. J. & Lea D. W. Effects of seawater carbonate ion concentration and temperature on shell U, Mg, and Sr in cultured planktonic foraminifera. Geochim. Cosmochim. Acta 68, 4347–4361 (2004).

[b22] Yu J., Elderfield H., Greaves M. & Day J. Preferential dissolution of benthic foraminiferal calcite during laboratory reductive cleaning. Geochem. Geophys. Geosyst. 8, Q06016 (2007).

[b23] Klinkhammer G. P. & Palmer M. R. Uranium in the oceans: where it goes and why. Geochim. Cosmochim. Acta 55, 1799–1806 (1991).

[b24] Froelich P. N. . Early oxidation of organic matter in pelagic sediments of the eastern equatorial Atlantic: suboxic diagenesis. Geochim. Cosmochim. Acta 43, 1075–1090 (1979).

[b25] Barnes C. E. & Cochran J. K. Uranium removal in oceanic sediments and the oceanic U balance. Earth Planet. Sci. Lett. 97, 94–101 (1990).

[b26] Boyle E. A. Manganese carbonate overgrowths on foraminifera tests. Geochim. Cosmochim. Acta 47, 1815–1819 (1983).

[b27] Sachs J. P. & Anderson R. F. Fidelity of alkenone paleotemperatures in southern Cape Basin sediment drifts. Paleoceanography 18, 1082 (2003).

[b28] Barker S. & Diz P. Timing of the descent into the last ice age determined by the bipolar seesaw. Paleoceanography 29, 489–507 (2014).

[b29] Emerson S., Fischer K., Reimers C. & Heggie D. Organic carbon dynamics and preservation in deep-sea sediments. Deep Sea Res. 32, 1–21 (1985).

[b30] McCorkle D. C. & Emerson S. R. The relationship between pore water carbon isotopic composition and bottom water oxygen concentration. Geochim. Cosmochim. Acta 52, 1169–1178 (1988).

[b31] Geslin E., Heinz P., Jorissen F. & Hemleben C. Migratory responses of deep-sea benthic foraminifera to variable oxygen conditions: laboratory investigations. Mar. Micropaleontol. 53, 227–243 (2004).

[b32] Duplessy J.-C. . ^13^C Record of benthic foraminifera in the last interglacial ocean: Implications for the carbon cycle and the global deep water circulation. Quat. Res. 21, 225–243 (1984).

[b33] Garcia H. E. . *World Ocean Atlas 2009* Vol. 3: Dissolved Oxygen, Apparent Oxygen Utilization, and Oxygen Saturation (Ed. Levitus, S.) 344 pp NOAA Atlas NESDIS 70, U.S. Government Printing Office, Washington, D.C. (2010).

[b34] Hodell D. A., Venz K. A., Charles C. D. & Ninnemann U. S. Pleistocene vertical carbon isotope and carbonate gradients in the South Atlantic sector of the Southern Ocean. Geochem. Geophys. Geosyst. 4, 1–19 (2003).

[b35] Schmiedl G. & Mackensen A. Late quaternary paleoproductivity and deep water circulation in the eastern South Atlantic Ocean: evidence from benthic foraminifera. Palaeogeogr. Palaeoclimatol. Palaeoecol. 130, 43–80 (1997).

[b36] Ninnemann U. S. & Charles C. D. Changes in the mode of Southern Ocean circulation over the last glacial cycle revealed by foraminiferal stable isotopic variability. Earth Planet. Sci. Lett. 201, 383–396 (2002).

[b37] Mackensen A., Rudolph M. & Kuhn G. Late Pleistocene deep-water circulation in the subantarctic eastern Atlantic. Glob. Planet. Change 30, 197–229 (2001).

[b38] Ragueneau O. . A review of the Si cycle in the modern ocean: recent progress and missing gaps in the application of biogenic opal as a paleoproductivity proxy. Glob. Planet. Change 26, 317–365 (2000).

[b39] Jaccard S. L., Galbraith E. D., Frölicher T. L. & Gruber N. Ocean (de)oxygenation across the last deglaciation: insights for the future. Oceanography 27, 26–35 (2014).

[b40] Anderson L. A. & Sarmiento J. L. Redfield ratios of remineralization determined by nutrient data analysis. Global Biogeochem. Cycles 8, 65–80 (1994).

[b41] Kwon E. Y., Sarmiento J. L., Toggweiler J. R. & DeVries T. The control of atmospheric pCO_2_ by ocean ventilation change: the effect of the oceanic storage of biogenic carbon. Global Biogeochem. Cycles 25, GB3026 (2011).

[b42] Curry W. B. & Oppo D. W. Glacial water mass geometry and the distribution of δ^13^C of ΣCO_2_ in the western Atlantic Ocean. Paleoceanography 20, PA1017 (2005).

[b43] Jaccard S. L. & Galbraith E. D. Large climate-driven changes of oceanic oxygen concentrations during the last deglaciation. Nat. Geosci. 5, 151–156 (2012).

[b44] Sarnthein M., Schneider B. & Grootes P. M. Peak glacial ^14^C ventilation ages suggest major draw-down of carbon into the abyssal ocean. Clim. Past 9, 2595–2614 (2013).

[b45] Schmittner A., Brook E. J. & Ahn J. in Ocean Circulation: Mechanisms and Impacts (eds. Schmittner A., Chiang J. C. H., Hemming S. R. 173, 209–246American Geophysical Union, Geophysical Monograph Series (2007).

[b46] Watson A. J. & Naveira Garabato A. C. The role of Southern Ocean mixing and upwelling in glacial-interglacial atmospheric CO_2_ change. Tellus B 58, 73–87 (2006).

[b47] Bereiter B. . Mode change of millennial CO_2_ variability during the last glacial cycle associated with a bipolar marine carbon seesaw. Proc. Natl. Acad. Sci. USA 109, 9755–9760 (2012). 22675123 10.1073/pnas.1204069109 PMC3382554

[b48] Jaccard S. L., Galbraith E. D., Martínez-Garcia A. & Anderson R. F. Covariation of abyssal Southern Ocean oxygenation and pCO_2_ throughout the last ice age. Nature 530, 207–210 (2016). 26840491 10.1038/nature16514

[b49] Sarmiento J. L., Gruber N., Brzezinski M. A. & Dunne J. P. High-latitude controls of thermocline nutrients and low latitude biological productivity. Nature 427, 56–60 (2004). 14702082 10.1038/nature02127

[b50] Carter L., McCave I. N. & Williams M. J. M. Circulation and water masses of the Southern Ocean: a review. Dev. Earth Environ. Sci. 8, 85–114 (2009).

[b51] Weiss R. F. The solubility of nitrogen, oxygen and argon in water and seawater. Deep Sea Res. 17, 721–735 (1970).

[b52] Adkins J. F., McIntyre K. & Schrag D. P. The salinity, temperature, and δ^18^O of the glacial deep ocean. Science 298, 1769–1773 (2002). 12459585 10.1126/science.1076252

[b53] Vogel H., Rosén P., Wagner B., Melles M. & Persson P. Fourier transform infrared spectroscopy, a new cost-effective tool for quantitative analysis of biogeochemical properties in long sediment records. J. Paleolimnol. 40, 689–702 (2008).

[b54] Meyer-Jacob C. . Independent measurement of biogenic silica in sediments by FTIR spectroscopy and PLS regression. J. Paleolimnol. 52, 245–255 (2014).

[b55] DeMaster D. J. The supply and accumulation of silica in the marine environment. Geochim. Cosmochim. Acta 45, 1715–1732 (1981).

[b56] Mortlock R. A. & Froelich P. N. A simple method for the rapid determination of biogenic opal in pelagic marine sediments. Deep Sea Res. 36, 1415–1426 (1989).

[b57] François R., Frank M., van der Loeff M. M. R. & Bacon M. P. ^230^Th normalization: an essential tool for interpreting sedimentary fluxes during the late Quaternary. Paleoceanography 19, 16 (2004).

[b58] Bourne M. D., Thomas A. L., Mac Niocaill C. & Henderson G. M. Improved determination of marine sedimentation rates using ^230^Th_xs_. Geochemistry Geophys. Geosystems 13, Q09017 (2012).

[b59] Vogel J. S., Southon J. R., Nelson D. E. & Brown T. A. Performance of catalytically condensed carbon for use in accelerator mass spectrometry. Nucl. Instrum. Methods Phys. Res. 5, 289–293 (1984).

[b60] Kuhn G. Susceptibility raw data of sediment core PS2498-1. http://dx.doi.org/10.1594/PANGAEA.87282 (2002).

[b61] Svensson A. . A 60000 year Greenland stratigraphic ice core chronology. Clim. Past 4, 47–57 (2008).

[b62] Veres D. . The Antarctic ice core chronology (AICC2012): an optimized multi-parameter and multi-site dating approach for the last 120 thousand years. Clim. Past 9, 1733–1748 (2013).

[b63] Key R. M. . A global ocean carbon climatology: Results from Global Data Analysis Project (GLODAP). Global Biogeochem. Cycles 18, GB4031 (2004).

[b64] Takahashi T. . Global sea-air CO_2_ flux based on climatological surface ocean pCO_2_, and seasonal biological and temperature effects. Deep Sea Res. 49, 1601–1622 (2002).

[b65] Orsi A. H., Whitworth T. & Nowlin W. D. On the meridional extent and fronts of the Antarctic Circumpolar Current. Deep Sea Res. 42, 641–673 (1995).

[b66] Lambert F. . Dust-climate couplings over the past 800,000 years from the EPICA Dome C ice core. Nature 452, 616–619 (2008). 18385736 10.1038/nature06763

[b67] Ahn J. & Brook E. J. Atmospheric CO_2_ and climate on millennial time scales during the last glacial period. Science 322, 83–85 (2008). 18787135 10.1126/science.1160832

[b68] Blunier T. & Brook E. J. Timing of millennial-scale climate change in Antarctica and Greenland during the last glacial period. Science 291, 109 (2001). 11141558 10.1126/science.291.5501.109

[b69] Lüthi D. . CO_2_ and O_2_/N_2_ variations in and just below the bubble-clathrate transformation zone of Antarctic ice cores. Earth Planet. Sci. Lett. 297, 226–233 (2010).

[b70] Monnin E. . Atmospheric CO_2_ concentrations over the last glacial termination. Science 291, 112 (2001). 11141559 10.1126/science.291.5501.112

[b71] Ahn J. & Brook E. J. Siple Dome ice reveals two modes of millennial CO_2_ change during the last ice age. Nat. Commun. 5, 3723 (2014). 24781344 10.1038/ncomms4723 PMC4015316

[b72] Indermühle A., Monnin E., Stauffer B., Stocker T. F. & Wahlen M. Atmospheric CO_2_ concentration from 60 to 20 kyr BP from the Taylor Dome ice core, Antarctica. Geophys. Res. Lett. 27, 735–738 (2000).

[b73] Marcott S. A. . Centennial-scale changes in the global carbon cycle during the last deglaciation. Nature 514, 616–619 (2014). 25355363 10.1038/nature13799

[b74] Ramsey C. B. . A complete terrestrial radiocarbon record for 11.2 to 52.8 kyr BP. Science 338, 370–374 (2012). 23087245 10.1126/science.1226660

[b75] Hughen K., Southon J., Lehman S., Bertrand C. & Turnbull J. Marine-derived ^14^C calibration and activity record for the past 50,000 years updated from the Cariaco Basin. Quat. Sci. Rev. 25, 3216–3227 (2006).

[b76] Reimer P. J. . IntCal09 and Marine09 radiocarbon age calibration curves, 0-50,000 years cal BP. Radiocarbon 51, 1111–1150 (2009).

[b77] Reimer P. J. . IntCal13 and Marine13 radiocarbon age calibration curves 0-50,000 years cal BP. Radiocarbon 55, 1869–1887 (2013).

