# Supplementary material for: Biological and physical controls in the Southern Ocean on past millennial-scale atmospheric CO2 changes
Source: Nat Commun. 2016 May 17;7:11539. doi: 10.1038/ncomms11539 (PMC4873644; doi:10.1038/ncomms11539)
Supplement: Supplementary Information — Supplementary Figures 1-8, Supplementary Tables 1-4 and Supplementary References. [file ncomms11539-s1.pdf]

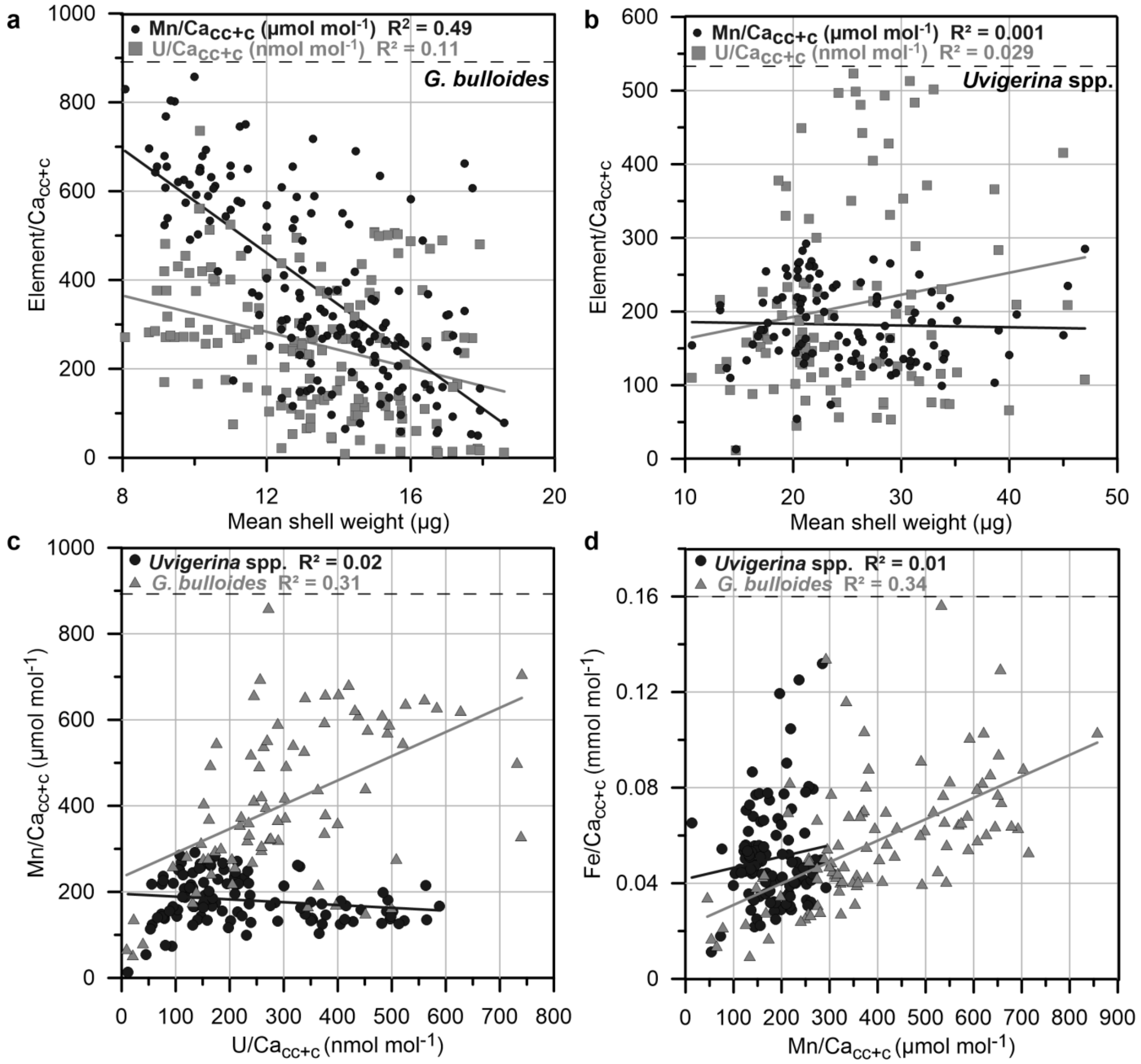

Supplementary Figure 1. Foraminifer shell weights and coating composition. Correlation of mean foraminiferal shell weights of (**a**) *G. bulloides* and (**b**) *Uvigerina* spp. with respective U/Ca_{cc+c} (grey) and Mn/Ca_{cc+c} (black) values, cross-plots of (**c**) Mn/Ca_{cc+c} versus U/Ca_{cc+c} as well as (**d**) Fe/Ca_{cc+c} versus Mn/Ca_{cc+c} of *Uvigerina* spp. (circles) and *G. bulloides* (triangles).

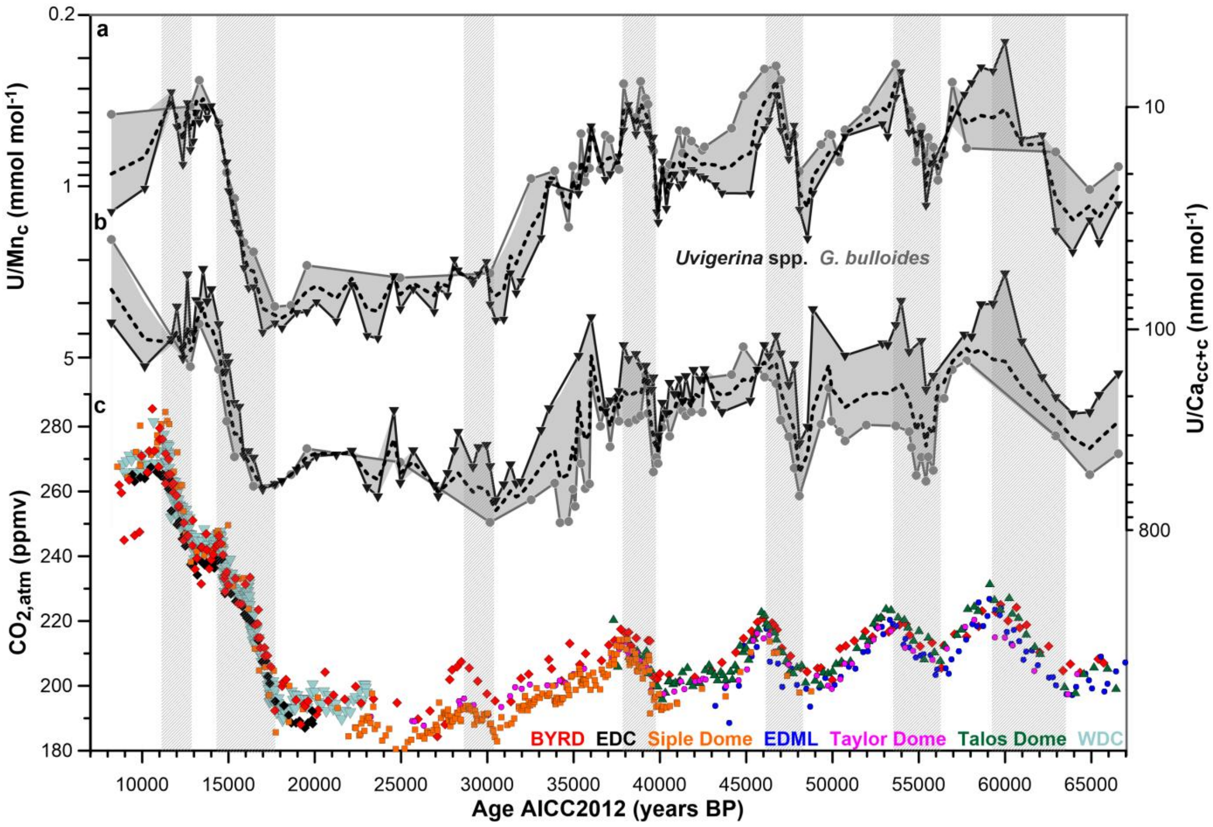

Supplementary Figure 2. Comparison of U/Mn$_c$ and U/Ca$_{cc+c}$. (**a**) U/Mn$_c$- and (**b**) U/Ca$_{cc+c}$ ratios obtained from *G. bulloides* (circles) and *Uvigerina* spp. (triangles) and their mean (stippled line); (**c**) variations in atmospheric CO_2 ($CO_{2,atm}$) recorded in the Antarctic ice cores BYRD[1,2], EDML[3,4], EDC[5], Taylor Dome[6], Siple Dome[7], Talos Dome[3] and WDC[8]. All data are shown on the AICC2012 age scale[9,10]. Highlighted areas indicate time intervals of rising $CO_{2,atm}$ concentrations.

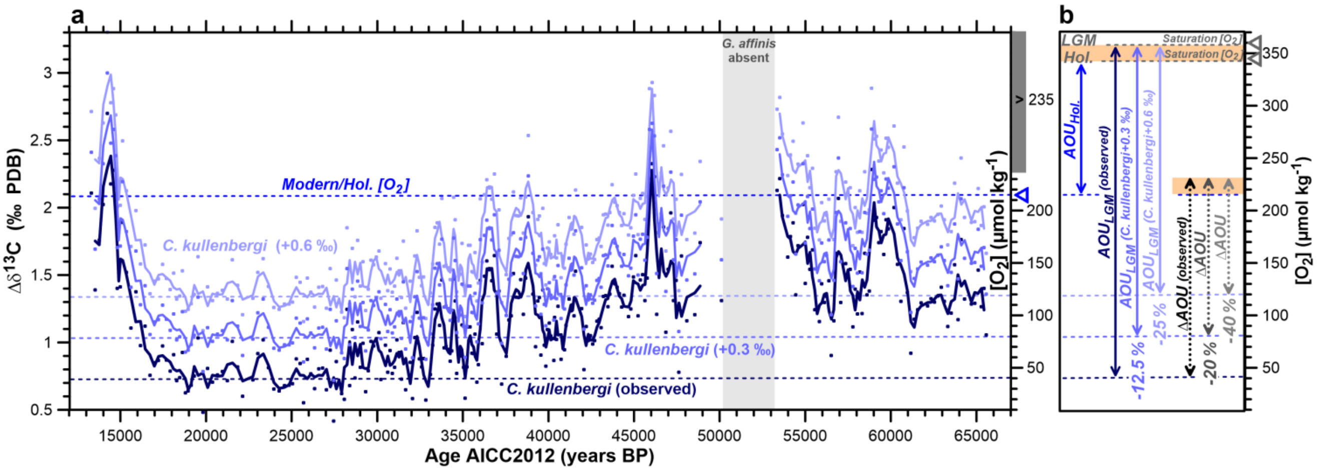

Supplementary Figure 3. Potential quantitative bias of $\Delta\delta^{13}C$-based bottom water $[O_2]$ reconstructions. (**a**) Bottom water $[O_2]$ and 'raw' $\delta^{13}C$ gradient between *C. kullenbergi* and *G. affinis* in sediment core MD07-3076Q (dark blue line, 3-pt running average) in comparison to the $\delta^{13}C$ gradient with adjusted *C. kullenbergi* $\delta^{13}C$ (+0.3 ‰ in blue and +0.6 ‰ in light blue; lines: 3-pt running averages). Triangle symbol on the right indicates modern bottom water $[O_2]$ at the core site of MD07-3076Q (~215 µmol mol^{-1})[11]. (**b**) Changes in the apparent oxygen utilization (AOU; $AOU=[O_2]_{Saturation}-[O_2]_{Reconstruction}$) during the last glacial maximum (LGM) and during the last deglaciation ($\Delta AOU=AOU_{LGM}-AOU_{Holocene}$) are illustrated for deviations of true glacial bottom water $\delta^{13}C$ from *C. kullenbergi* $\delta^{13}C$ and associated changes in absolute glacial bottom water $[O_2]$ in the deep sub-Antarctic Atlantic. Grey triangles on the right indicate modern saturation $[O_2]$ at the core site (~345 µmol mol^{-1})[11] and saturation $[O_2]$ estimated for the LGM (Methods). The difference between both is highlighted as orange bar; Hol.-Holocene.

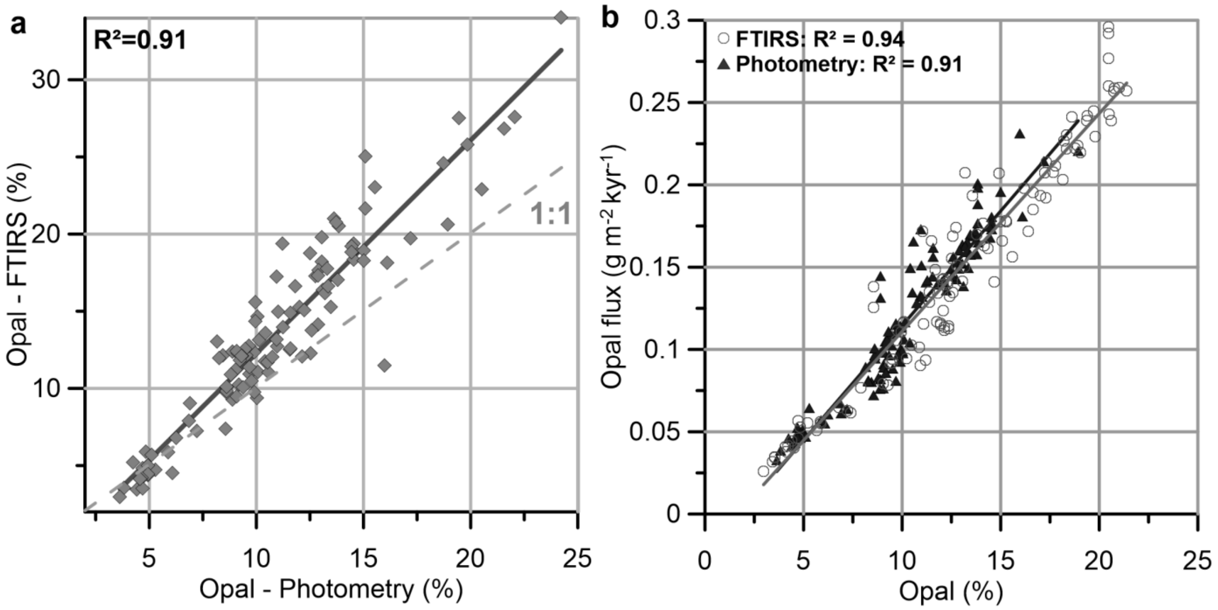

Supplementary Figure 4. Export productivity variations inferred from MD07-3076Q sediments. (**a**) Photometrically determined sedimentary opal content versus Fourier Transform Infrared Spectroscopy (FTIRS)-based independently calibrated sedimentary opal content measurements; (**b**) sedimentary opal content determined by means of FTIRS (circles) and photometry (triangles) versus respective ^{230}Thorium-normalized opal fluxes.

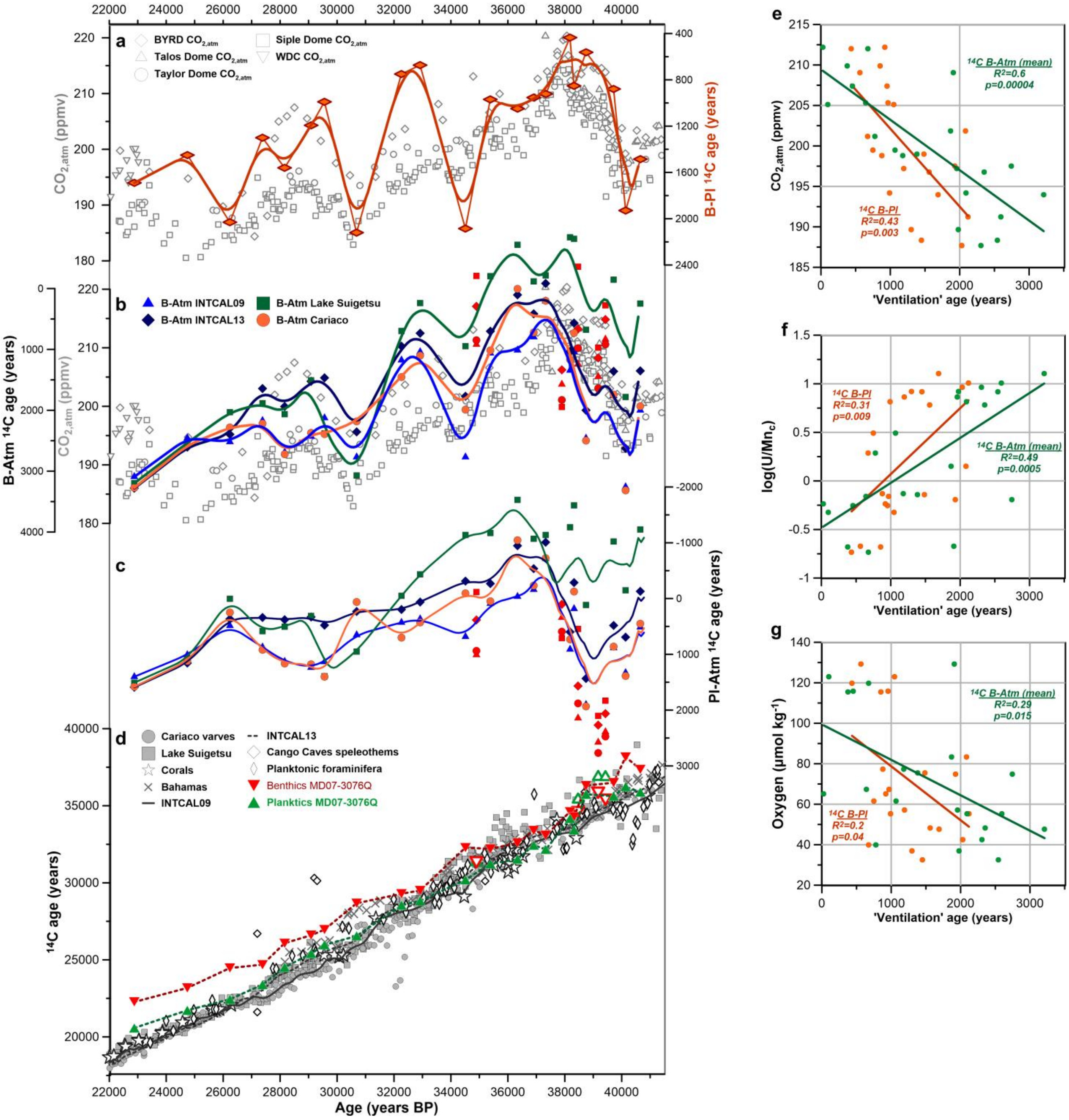

Supplementary Figure 5. Benthic and planktonic ^{14}C ages in MD07-3076Q in comparison to atmospheric ^{14}C and CO_2. (**a**) B-Pl ^{14}C age offsets (orange) plotted on top of $CO_{2,atm}$ recorded in the Antarctic ice cores (grey, refs. as in Supplementary Figure 2) shown on the AICC2012 age scale[9,10], (**b**) B-Atm ^{14}C age offsets based on different calibration datasets, i.e. Intcal09 (blue)[12], Intcal13 (dark blue)[13], Lake Suigetsu (green)[14] and Cariaco Basin (orange)[15], shown as 1,000 years-running means (red symbols indicate samples where benthic foraminifera are younger than co-existing planktonic foraminifera), plotted on top of $CO_{2,atm}$ (grey), (**c**) planktonic foraminifer ^{14}C age offsets from different atmospheric ^{14}C curves (as in b), (**d**) atmospheric ^{14}C ages (datasets as in b, including Bahamas speleothems[16],

Cango Cave speleothems[17], planktonic foraminifera from Iberian margin sediment cores[18,19] and corals[20]) in comparison to benthic (red) and planktonic ^{14}C ages (green) obtained in MD07-3076Q sediments (open symbols refer to those samples, where planktonic foraminifer samples are found to be older than benthic foraminifer samples); cross-plots of B-Pl ^{14}C and B-Atm ^{14}C age offsets with (**e**) $CO_{2,atm}$, (**f**) the logarithm of mean foraminifer (i.e. *G. bulloides* and *Uvigerina* spp.) coating U/Mn ratios (stippled line in Supplementary Figure 2a) and (**g**) reconstructed bottom water oxygen levels.

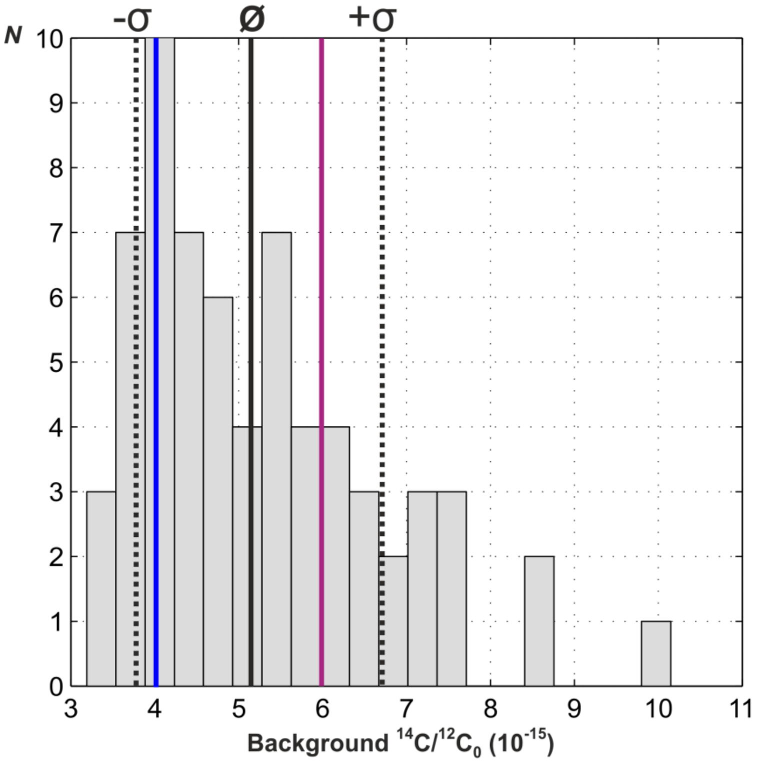

Supplementary Figure 6. Radiocarbon backgrounds. Histogram of the radiocarbon content of radiocarbon-dead carbonate material used for background corrections of foraminifer samples graphitized in the Godwin Radiocarbon Laboratory (April 2011 - January 2015). Purple and blue line show background corrections applied to our data in Supplementary Figure 7.

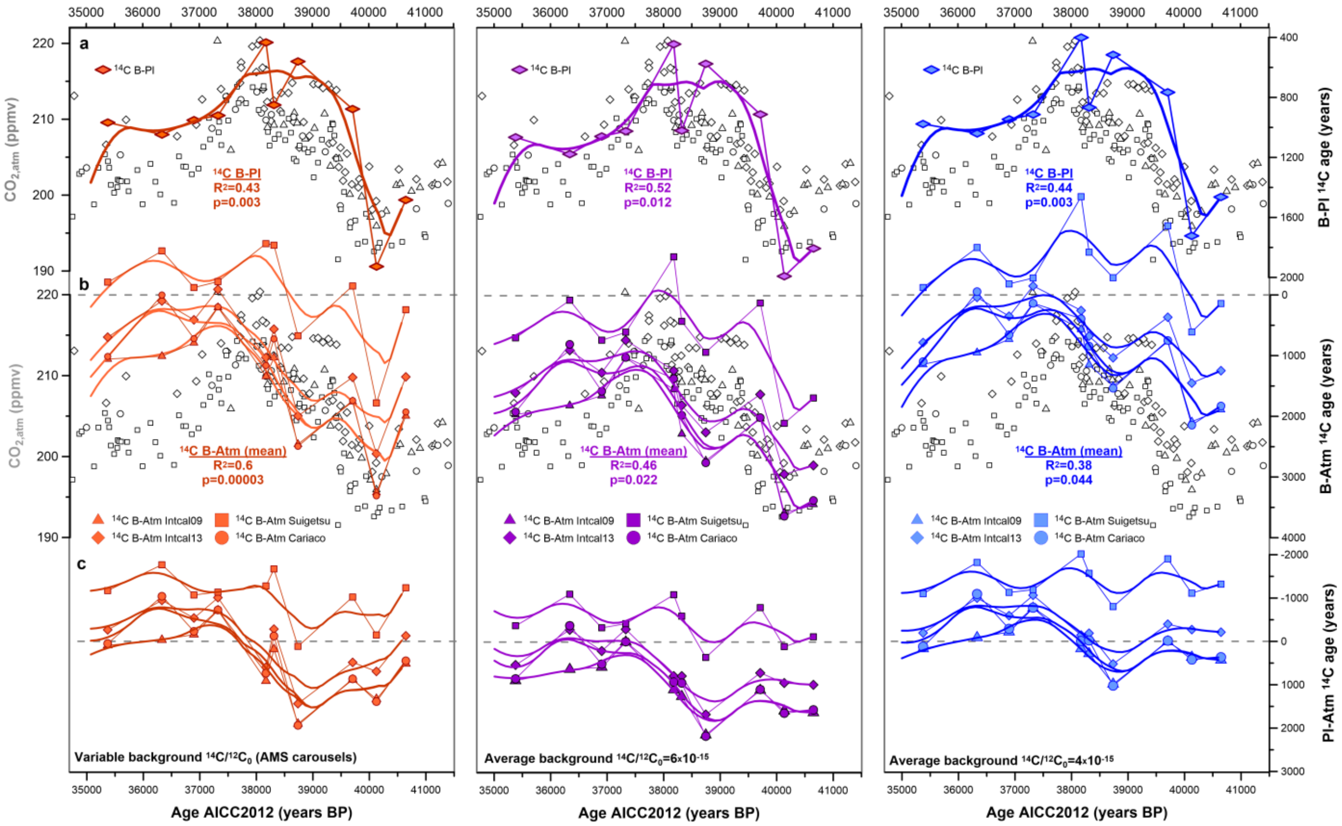

Supplementary Figure 7. Effect of background corrections on B-Pl ^{14}C age offsets, B-Atm ^{14}C age offsets and surface ocean reservoir ages (Pl-Atm ^{14}C age offsets). (**a**) B-Pl ^{14}C age offsets (colored symbols) and (**b**) B-Atm ^{14}C age offsets (colored symbols) plotted on top of variations in $CO_{2,atm}$ measured in Antarctic ice cores (open symbols, refs. as in Supplementary Figure 2) as well as (**c**) Pl-Atm ^{14}C age offsets (colored symbols) for background corrections based on the individual AMS carousel background measurements of radiocarbon-dead spar calcite (left, Supplementary Table 4), a hypothetic true absolute background of $^{14}C/^{12}C_0=6x10^{-15}$ (middle) and $^{14}C/^{12}C_0=4x10^{-15}$ (right) that are within one-sigma uncertainty of the Godwin Radiocarbon Laboratory-internal background mean (Supplementary Figure 6). Thick lines show 1 kyr-averages.

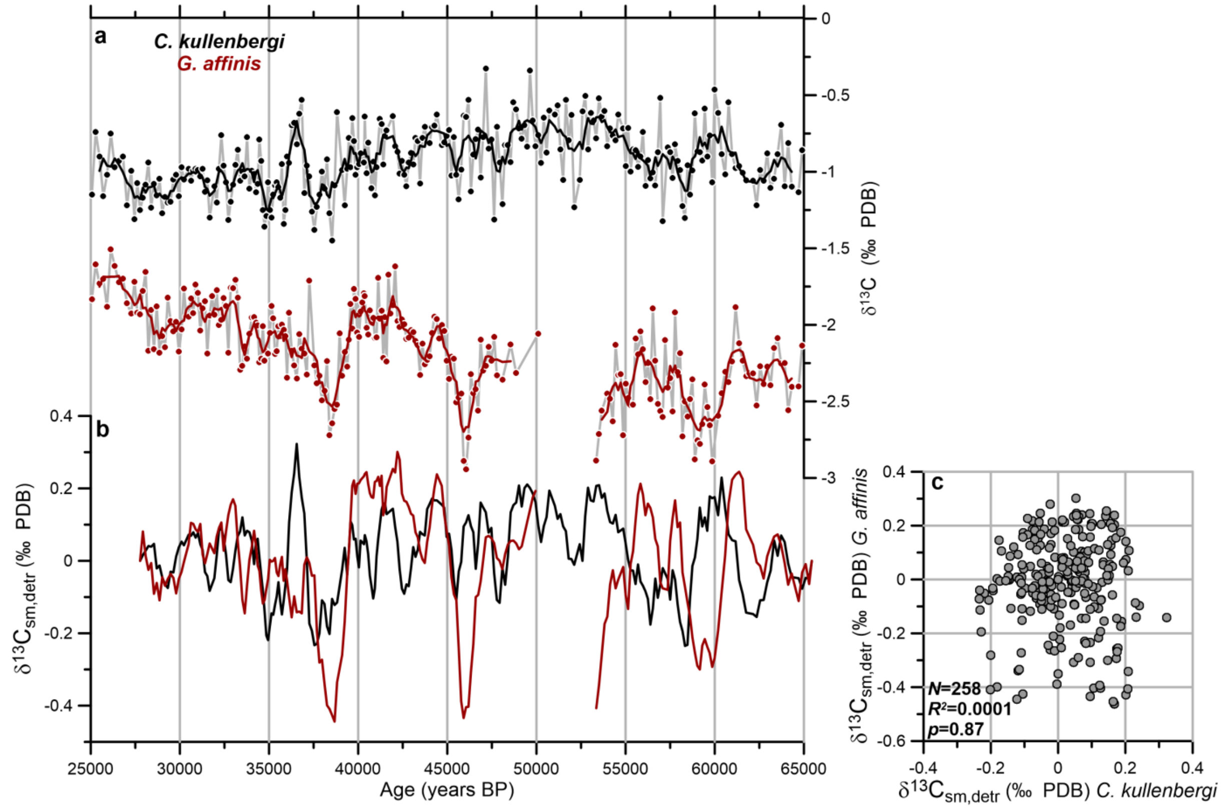

Supplementary Figure 8. ***C. kullenbergi*** **δ^{13}C and** ***G. affinis*** **δ^{13}C in MD07-3076Q**. (**a**) Raw benthic δ^{13}C (solid line shows 3-point running averages); (**b**) detrended ('detr'; via the subtraction of the long-term 8 kyr- running mean) and smoothed benthic δ^{13}C ('sm'; using a 500 year- sliding window), (**c**) cross-plot of the detrended and smoothed *C. kullenbergi* and *G. affinis* δ^{13}C records in MD07-3076Q shows a poor correlation.

Supplementary Table 1. Analytical parameter setting of the iCAP-Q ICP-MS applied for determining the abundance of U- and Th isotopes in MD07-3076Q sediment samples

Desolvator	APEX HF
Nebulizer, flow rate	Self-aspirating microflow PFA nebulizer: 100 µl/min
Cones	sample and skimmer: Ni
Power	1548 W
Gas flows Cool gas:	13.8 l/min
Auxiliary gas:	0.78 l/min
Sample gas:	1.02 l/min
measurement mode	STDS
Sensitivity (^{238}U) Standard:	1,600 kcps/ppb
Background with 0.5 N HNO_3	^{229}Th: ≤ 0.5 cps<br>^{230}Th: ≤ 0.7 cps<br>^{232}Th: ≤ 5000 cps<br>$^{235,236}U$: ≤ 3 cps<br>^{238}U: ≤ 3000 cps
Oxides ($^{238}U.^{16}O/^{238}U$)	≤ 3 %
Abundance sensitivity	mass237/mass238 < $1.6x10^{-4}$
Rinsing	Th: 7 Min 1M HNO_3 + 0.01 M HF<br>U: 2 Min 1M HNO_3
Typical blank contribution on<br>^{230}Th<br>^{232}Th<br>^{238}U<br>^{234}U	 <0.1 %<br><0.05 %<br><1 %<br><1 %
Average full analytical reproducibility (replicates n=5)	^{230}Th: 2.8 %<br>^{232}Th: 2.3 %<br>^{238}U: 0.4 %<br>^{234}U: 0.5 %

Supplementary Table 2. Procedural specifications of U- and Th measurements with the iCAP-Q ICP-MS at the University of Heidelberg

Scanned mass (u)	229	230	232	233	234	235	236
Dwell time (s)	0.05	0.05	0.001	0.03	0.03	0.01	0.01
Channels	7	7	5	3	3	3	3
Cycles	35	35	35	35	35	35	35
Sweeps	15	15	15	20	20	20	20
Spacing (u)	0.1	0.1	0.1	0.1	0.1	0.1	0.1

Supplementary Table 3. New planktonic and benthic ^{14}C measurements in sediment core MD07-3076Q

Depth (cm)	Calendar age (years BP)	*N. pachyderma* (s.) ^{14}C age (^{14}C years)	±σ *N. pachyderma* (s.) ^{14}C age (^{14}C years)	Mixed benthic ^{14}C age (^{14}C years)	±σ Mixed benthic ^{14}C age (^{14}C years)	B-Pl ^{14}C age offset (^{14}C years)
219.5	29090	25407	85	26600	130	1193
265.5	32259	28540	89	29290	160	750
275.5	32915	28827	91	29500	190	673
301.5	34508	30195	411	32280	490	2085
319.5	35382	31235	316	32204	389	969
337.5	36341	31533	394	32582	452	1049
345.5	36907	32447	438	33400	522	953
351.5	37332	32159	463	33081	479	922
363.5	38181	34174	758	34609	804	435
365.5	38322	33429	421	34281	474	852
371.5	38747	35747	926	36309	1010	562
387.5	39713	35620	916	36499	1042	879
396.5	40137	36236	986	38165	1277	1929
407.5	40654	35879	680	37364	830	1485

Supplementary Table 4. Last glacial B-Atm ^{14}C (ventilation) ages and Pl-Atm ^{14}C (surface ocean reservoir) ages in sediment core MD07-3076Q from this study and ref. 21

		^{14}C B-Atm (^{14}C years)						^{14}C Pl-Atm (^{14}C years)					
Depth (cm)	Cal. age (years BP)	IntCal09	IntCal13	Lake Suigetsu	Cariaco	mean	±σ	IntCal09	IntCal13	Lake Suigetsu	Cariaco	mean	±σ
161.5	22881	3091	3281	3198	3270	3210	95	1401	1591	1508	1580	1520	95
173.5	24743	2458	2605	2535	2573	2543	77	1008	1155	1085	1123	1093	77
187.5	26234	2517	2394	2033	2282	2307	241	487	364	3	252	277	241
197.5	27380	2169	1641	1885	2219	1979	321	869	341	585	919	679	321
207.5	28157	2689	1938	2064	2725	2133	716	1129	378	504	1165	573	716
219.5	29090	2430	1522	1507	2371	1714	830	1237	329	314	1178	521	830
225.5	29557	2126	1467	2381	2390	1834	830	1136	477	1391	1400	844	830
241.5	30685	2770	2354	3071	2182	2172	1233	650	234	951	62	52	1233
265.5	32259	1177	946	699	1453	1058	413	427	196	-51	703	308	413
275.5	32915	1040	734	239	1102	678	459	367	61	-434	429	5	459
301.5	34508	2770	1771	946	1990	1873	873	685	-314	-1139	-95	-212	873
319.5	35382	1060	700	-205	1016	595	666	91	-269	-1174	47	-374	666
337.5	36341	1013	102	-720	6	33	795	-36	-947	-1769	-1043	-1016	795
345.5	36907	794	415	-118	723	395	482	-159	-538	-1071	-230	-558	482
351.5	37332	208	-86	-216	200	-22	272	-714	-1008	-1138	-722	-944	272
363.5	38181	1345	1033	-842	1168	727	899	910	598	-1277	733	292	899
365.5	38322	1035	566	-817	729	379	955	183	-286	-1669	-123	-473	955
371.5	38747	2462	1997	677	2497	1978	915	1900	1435	115	1935	1416	915
387.5	39713	1763	1361	-145	1743	1219	930	884	482	-1024	864	340	930
396.5	40137	3261	2623	1783	3316	2668	803	1332	694	-146	1387	739	803
407.5	40654	1997	1354	246	1934	1345	881	512	-131	-1239	449	-140	861

Supplementary References

1. Ahn, J. & Brook, E. J. Atmospheric CO_2 and climate on millennial time scales during the last glacial period. *Science* **322,** 83–85 (2008).
2. Blunier, T. & Brook, E. J. Timing of millennial-scale climate change in Antarctica and Greenland during the last glacial period. *Science* **291,** 109–112 (2001).
3. Bereiter, B. *et al.* Mode change of millennial CO_2 variability during the last glacial cycle associated with a bipolar marine carbon seesaw. *Proc. Natl. Acad. Sci.* **109,** 9755–9760 (2012).
4. Lüthi, D. *et al.* CO_2 and O_2/N_2 variations in and just below the bubble-clathrate transformation zone of Antarctic ice cores. *Earth Planet. Sci. Lett.* **297,** 226–233 (2010).
5. Monnin, E. *et al.* Atmospheric CO_2 concentrations over the last glacial termination. *Science* **291,** 112–114 (2001).
6. Indermühle, A., Monnin, E., Stauffer, B., Stocker, T. F. & Wahlen, M. Atmospheric CO_2 concentration from 60 to 20 kyr BP from the Taylor Dome ice core, Antarctica. *Geophys. Res. Lett.* **27,** 735–738 (2000).
7. Ahn, J. & Brook, E. J. Siple Dome ice reveals two modes of millennial CO_2 change during the last ice age. *Nat. Commun.* **5,** 3723 (2014).
8. Marcott, S. A. *et al.* Centennial-scale changes in the global carbon cycle during the last deglaciation. *Nature* **514,** 616–619 (2014).
9. Veres, D. *et al.* The Antarctic ice core chronology (AICC2012): an optimized multi-parameter and multi-site dating approach for the last 120 thousand years. *Clim. Past* **9,** 1733–1748 (2013).
10. Gottschalk, J., Skinner, L. C. & Waelbroeck, C. Contribution of seasonal sub-Antarctic surface water variability to millennial-scale changes in atmospheric CO_2 over the last deglaciation and Marine Isotope Stage 3. *Earth Planet. Sci. Lett.* **411,** 87–99 (2015).
11. Garcia, H. E. *et al. World Ocean Atlas 2009, Volume 3: Dissolved Oxygen, Apparent Oxygen Utilization, and Oxygen Saturation.* **3,** National Oceanic and Atmospheric Administration, (2010).
12. Reimer, P. J. *et al.* IntCal09 and Marine09 radiocarbon age calibration curves, 0-50,000 years cal BP. *Radiocarbon* **51,** 1111–1150 (2009).
13. Reimer, P. J. *et al.* IntCal13 and Marine13 radiocarbon age calibration curves 0-50,000 years cal BP. *Radiocarbon* **55,** 1869–1887 (2013).
14. Ramsey, C. B. *et al.* A complete terrestrial radiocarbon record for 11.2 to 52.8 kyr BP. *Science* **338,** 370–374 (2012).
15. Hughen, K., Southon, J., Lehman, S., Bertrand, C. & Turnbull, J. Marine-derived ^{14}C calibration and activity record for the past 50,000 years updated from the Cariaco Basin. *Quat. Sci. Rev.* **25,** 3216–3227 (2006).
16. Hoffmann, D. L. *et al.* Towards radiocarbon calibration beyond 28 ka using speleothems from the Bahamas. *Earth Planet. Sci. Lett.* **289,** 1–10 (2010).
17. Vogel, J. C. & Kronfeld, J. Calibration of radiocarbon dates for the late Pleistocene using U/Th dates on stalagmites. *Radiocarbon* **39,** 27–32 (2006).
18. Bard, E., Rostek, F. & Ménot-Combes, G. Radiocarbon calibration beyond 20,000 ^{14}C yr BP by means of planktonic foraminifera of the Iberian Margin. *Quat. Res.* **61,** 204–214 (2004).
19. Völker, A. L. *et al.* Correlation of marine ^{14}C ages from the Nordic Seas with the GISP2 isotope record: implications for ^{14}C calibration beyond 25 ka BP. *Radiocarbon* **40,** 517–534 (2006).
20. Fairbanks, R. G. *et al.* Radiocarbon calibration curve spanning 0 to 50,000 years BP based on paired $^{230}Th/^{234}U/^{238}U$ and ^{14}C dates on pristine corals. *Quat. Sci. Rev.* **24,** 1781–1796 (2005).
21. Skinner, L. C., Fallon, S., Waelbroeck, C., Michel, E. & Barker, S. Ventilation of the deep Southern Ocean and deglacial CO_2 rise. *Science* **328,** 1147–1151 (2010).
